# Supplementary material for: Codesign and development of a primary school based pathway for child anxiety screening and intervention delivery: a protocol, mixed-methods feasibility study
Source: BMJ Open. 2021 Apr 20;11(4):e044852. doi: 10.1136/bmjopen-2020-044852 (PMC8061838; doi:10.1136/bmjopen-2020-044852)
Supplement: Author's manuscript [file bmjopen-2020-044852.draft_revisions.pdf]

BMJ Open is committed to open peer review. As part of this commitment we make the peer review history of every article we publish publicly available.

When an article is published we post the peer reviewers' comments and the authors' responses online. We also post the versions of the paper that were used during peer review. These are the versions that the peer review comments apply to.

The versions of the paper that follow are the versions that were submitted during the peer review process. They are not the versions of record or the final published versions. They should not be cited or distributed as the published version of this manuscript.

BMJ Open is an open access journal and the full, final, typeset and author-corrected version of record of the manuscript is available on our site with no access controls, subscription charges or pay-per-view fees (http://bmjopen.bmj.com).

If you have any questions on BMJ Open's open peer review process please email info.bmjopen@bmj.com

BMJ Open

Protocol for the co-design and development of a school-based pathway for child anxiety screening and intervention delivery

Journal:	*BMJ Open*
Manuscript ID	bmjopen-2020-044852
Article Type:	Protocol
Date Submitted by the Author:	16-Sep-2020

Complete List of Authors:	Williamson, Victoria; King's College London; Oxford University<br>Larkin, Michael; Aston University<br>Reardon, Tessa; Oxford University<br>Pearcey, Samantha; Oxford University<br>Hill, Claire; University of Reading<br>Stallard, Paul; University of Bath<br>Spence, Susan; Griffith University<br>Macdonald, Ian; Charlie Waller Institute<br>Ukoumunne, Obioha; University of Exeter Medical School, NIHR CLAHRC South West Peninsula (PenCLAHRC)<br>Creswell , Cathy ; University of Oxford,

Keywords:	Anxiety disorders < PSYCHIATRY, Protocols & guidelines < HEALTH SERVICES ADMINISTRATION & MANAGEMENT, MENTAL HEALTH, Child & adolescent psychiatry < PSYCHIATRY, QUALITATIVE RESEARCH

SCHOLARONE™
Manuscripts

I, the Submitting Author has the right to grant and does grant on behalf of all authors of the Work (as defined in the below author licence), an exclusive licence and/or a non-exclusive licence for contributions from authors who are: i) UK Crown employees; ii) where BMJ has agreed a CC-BY licence shall apply, and/or iii) in accordance with the terms applicable for US Federal Government officers or employees acting as part of their official duties; on a worldwide, perpetual, irrevocable, royalty-free basis to BMJ Publishing Group Ltd ("BMJ") its licensees and where the relevant Journal is co-owned by BMJ to the co-owners of the Journal, to publish the Work in this journal and any other BMJ products and to exploit all rights, as set out in our licence.

The Submitting Author accepts and understands that any supply made under these terms is made by BMJ to the Submitting Author unless you are acting as an employee on behalf of your employer or a postgraduate student of an affiliated institution which is paying any applicable article publishing charge ("APC") for Open Access articles. Where the Submitting Author wishes to make the Work available on an Open Access basis (and intends to pay the relevant APC), the terms of reuse of such Open Access shall be governed by a Creative Commons licence – details of these licences and which Creative Commons licence will apply to this Work are set out in our licence referred to above.

Other than as permitted in any relevant BMJ Author's Self Archiving Policies, I confirm this Work has not been accepted for publication elsewhere, is not being considered for publication elsewhere and does not duplicate material already published. I confirm all authors consent to publication of this Work and authorise the granting of this licence.

For peer review only

Protocol for the co-design and development of a school-based pathway for child anxiety screening and intervention delivery

Williamson, V.[a,b*], Larkin, M.[c] , Tessa Reardon[a] , Samantha Pearcy[a], Claire Hill[d], Paul Stallard[e], Sue Spence[f], Maria Breen[d], Ian Mcdonald[g], ObiohaUkoumunne[d,], Creswell, C. [a]

[a] Department of Experimental Psychology, Anna Watts Building, University of Oxford, Oxford, OX2 6 GG

[b]Institute of Psychiatry, Psychology and Neuroscience, King's College London, London, SE5 9RJ

[c] Aston Neuroscience Institute, Department of Psychology, Aston University, Birmingham, B4 7ET

[d] School of Psychology & Clinical Language Sciences NIHR ARC South West Peninsula, University of Reading, Reading, RG6 6ALExeter, Exeter, EX1 2LU

[e] University of Bath, Claverton Down, Bath BA2 7AY

[f] Griffith University, 16 Russell Street South Bank, QLD 4101, AU

[g] Charlie Waller Memorial Trust, First Floor, Rear Office, 32 High Street, Thatcham RG19 3JD

***Correspondence:** Dr Victoria Williamson, Department of Experimental Psychology, University of Oxford, Oxford, OX2 6 GG; Institute of Psychiatry, Psychology and Neuroscience,

For peer review only

King’s College London, London, SE5 9RJ.

Victoria.williamson@kcl.ac.uk

Funding: This paper represents independent research funded by the National Institute for Health Research (NIHR; RP-PG-0218-20010) (CC). The views expressed are those of the authors and not necessarily those of the NHS, the NIHR or the Department of Health and Social Care.

Conflicts of interest: Authors have no conflicts of interest to declare.

Author contributions: All authors contributed towards the design of the study protocol, contributed towards the writing of the manuscript and approved the final version of the protocol

Word Count: 3410

For peer review only

Abstract

Introduction: Anxiety disorders are among the most common mental health problems in childhood. Despite this, few children access evidence-based interventions and school may be an ideal setting to improve children's access to treatment. This paper describes the design, sampling methods, and expected data collection of the Identifying Child Anxiety Though Schools – Identification to Intervention (iCATS i2i) study which aims to develop acceptable school-based procedures to identify and support child anxiety difficulties.

Methods and analysis: iCATS i2i will use a mixed-methods approach to co-design and deliver a set of procedures – or 'pathway' – to improve access to evidence-based intervention for child anxiety difficulties through primary schools in England. The study will consist of four stages, initially involving in-depth interviews with parents, children, school staff, stakeholders (Stage 1) to inform the development of the pathway. The pathway will then be administered in two primary schools, including screening, feedback to parents, and the offer of treatment where indicated (Stage 2), with participating children, parents and school staff invited to provide feedback on their experience (Stages 3 & 4). Data will be analysed using Template Analysis.

Ethics and dissemination: The iCATS i2i study was approved by the University of Oxford's Research Ethics Committee (REF R64620/RE001). It is expected that this co-design study will lead on to a future

For peer review only

feasibility study and, if indicated, a randomised controlled trial. By providing information on child, parent, school-staff and other stakeholder's experiences, we anticipate that the findings will inform the development of an acceptable evidence-based pathway for identification and intervention for children with anxiety disorders in primary schools and may also inform broader approaches to screening for and treating youth mental health problems outside of clinics.

Strengths and limitations of this study:

- Focus on child anxiety disorders, one of the most common mental helath problems in childhood.
- By using a co-design approach which incorporates feedback from children, parents, school-staff and stakeholders, this study will lead to the development of acceptable procedures for screening and offering treatment for child anxiety difficulties in primary schools.
- The study is limited by the use of an 'opt-in' approach to consent which could introduce participation bias.
- The primary use of online platforms for consent, screening and delivery of the CBT intervention may exclude families who have limited access to technology or lack technical skills; although ways to facilitate the participation of those in these situations will be explored.

For peer review only - http://bmjopen.bmj.com/site/about/guidelines.xhtml

For peer review only

Introduction

Anxiety disorders are among the most common mental health problems in childhood (6.5% prevalence; [1]) and approximately half of all anxiety disorders emerge by the age of 11 years. Childhood anxiety disorders are often chronic and pervasive, and have an adverse effect on social, education and familial functioning. Childhood anxiety disorders often persist into adulthood when left untreated [2] and are associated with comorbid mental health difficulties, including major depression and substance abuse [3,4]. The societal cost of a child with an anxiety disorder is estimated to be 21 times that of a non-anxious child [5]. As such, effective identification and treatment of anxiety disorders in childhood is important.

Effective treatments for childhood anxiety disorders exist. However, very few children are offered or are able to access them [6,7]. For example, previous research has shown that only 2% of pre-adolescent children who meet criteria for an anxiety disorder in England receive an evidence-based intervention [7]. Barriers to receiving evidence-based treatment include difficulties with identification of anxiety difficulties, concerns regarding stigma to the child or family, and long waiting lists at Child and Adolescent Mental Health Services (CAMHS)[7]. In recent years, increasing evidence for the effectiveness of online, remotely delivered treatments for childhood anxiety difficulties has been found

For peer review only - http://bmjopen.bmj.com/site/about/guidelines.xhtml

and the delivery of online treatment may overcome some of these barriers to care [8]. The vast majority of children attend school [9] where they spend much of their time, therefore schools are also an ideal setting to overcome many of these barriers. However, there are not clear mechanisms for identifying youth mental health difficulties in schools nor clear pathways for those who would benefit to access evidence-based treatments.

One approach often used in healthcare service design and development is 'co-design', where the knowledge and lived experiences of service users themselves are drawn upon to enhance the quality and experiences of care [10,11]. Co-design aims to develop an in-depth understanding of how stakeholders and service users perceive and experience the look, feel, procedures and structures of a service [12]. By engaging stakeholders and service users in co-designing a service, this is thought to lead to better quality of care and improved service performance by highlighting individual's subjective feelings at various points in the care pathway, which, in turn, may lead to improvements in health outcomes and more efficient allocation of limited healthcare resources [13]. Given the importance of early intervention, an acceptable pathway that effectively identifies anxious children and successfully increases access to evidence-based treatment is urgently needed. The Identifying Child Anxiety Though Schools – Identification to Intervention (iCATS i2i) study will

For peer review only - http://bmjopen.bmj.com/site/about/guidelines.xhtml

develop procedures to identify and support child anxiety difficulties through schools informed by a co-design approach.

This article describes the iCATS i2i co-design protocol. The co-designed procedures will be evaluated in a subsequent feasibility study and, if indicated, randomised control trial beginning in 2021.

Method

This protocol and associated procedures were approved by the Central University Research Ethics Committee at the University of Oxford (REF R64620/RE001).

Study design

We will apply a mixed-methods approach to co-design, produce and deliver a set of procedures – or 'pathway' – to improve access to evidence-based intervention for child anxiety difficulties through primary schools in England. Several of the key elements of the pathway were specified in advance of the co-design work based on the existing empirical literature and parent and school staff consultation. Specifically, it was pre-specified that children's anxiety difficulties would be screened using questionnaire measures, parents would receive feedback, and, where indicated, a brief online treatment for child anxiety difficulties would be offered.

In parallel to this work we are working on refining measures for screening for child anxiety problems (Reardon et al. under review), but in the interim we will screen using

For peer review only - http://bmjopen.bmj.com/site/about/guidelines.xhtml

brief child, parent and teacher versions of the Spence Child Anxiety Scale (SCAS-8;[14]) together with four items that assess the extent of interference in everyday life generated to assess the impact and chronicity of and perceived need for help for anxiety difficulties. We will consider a child to have screened 'positive' for likely anxiety difficulties if they score above the cut off on the SCAS-8 on the basis of any reporter (score of 7.5 for parents, 6.5 for children, 4.5 for teachers) and/or indicate that anxiety interferes at least 1 'only a little' on any of the interferance items. This interference-based cut off score was based on feedback from the dedicated stakeholder group. The use of a screening questionnaire to determine which children may benefit from additional support with anxiety was a prespecified component of the study as this approach shows promise for increasing access to support (e.g. [15]).

The treatment to be offered is an online version of a brief therapist-guided parent-delivered CBT approach for child anxiety disorders (OSI; Online Support and Intervention for child anxiety). OSI was originally developed for use in National Health Service (NHS) clinics and was co-designed by NHS clinicians, parents and children who had received treatment for anxiety (Hill et al., in preparation). This treatment was selected as it is brief, effective [16] and more cost-effective than brief face-to-face psychological therapy [17] and can be delivered by non-expert practitioners (e.g.

For peer review only - http://bmjopen.bmj.com/site/about/guidelines.xhtml

[18]). The approach of working directly with the parent, rather than the child, also addressed particular barriers to seeking and accessing help for anxiety highlighted by parents, including the preference to be supported to manage the difficulties as a family and for the child not to be singled out [7]. The online version of this intervention involves 7 online modules for parents, supported by a weekly 20-minute telephone call with a Children's Wellbeing Practitioner (CWP, NHS Band 5), with a follow-up telephone session 4-weeks later. Modules teach parents how to explore their child's anxious thoughts, put them to the test through facing fears, and to problem solve challenges that arise. This is accompanied by a game app for the child to help motivate them to face their fears.

We will use a mixed-method co-design process to determine *how* the pre-specified parts of the pathway should be presented, who by, and to address any important considerations to optimise accessibility of and engagement with the pathway. The co-design process will consist of four stages (see Figure 1) involving initial interviews and focus groups with parents, children, school staff, stakeholders (Stage 1) to inform the development of a set of procedures that will be applied in two schools. These procedures will be delivered with participating children, parents and school staff (Stage 2) who will provide feedback on their experience (Stage 3 & 4), including cued-recall specifically on the experience of receiving feedback on

For peer review only - http://bmjopen.bmj.com/site/about/guidelines.xhtml

whether their child experiences difficulties with anxiety. Feedback from those families who choose not to be involved in the study or dropped out will also be sought to ensure any barriers to engagement are captured (Stage 4).

.

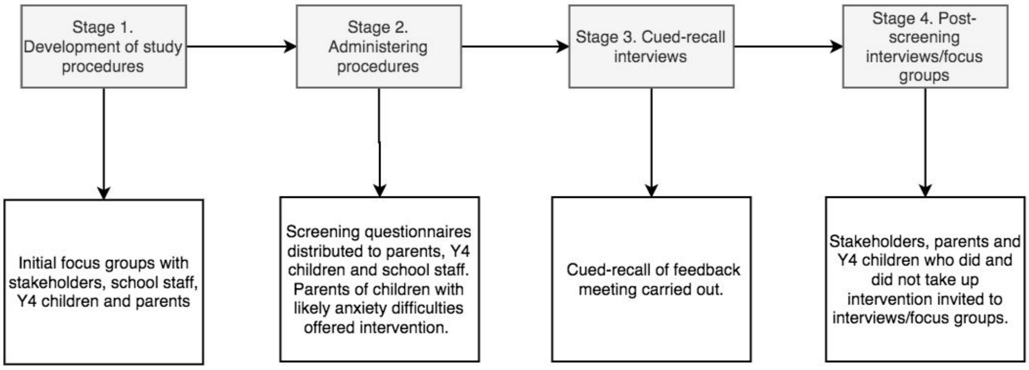

Figure 1. Overview of the co-design process for developing the iCATS i2i protocol

Note. Y4 = year four.

Patient and public involvement (PPI)

Involvement from parents, school staff and wider stakeholders informed the development of this protocol, the pre-specified elements of the pathway and will contribute throughout the delivery of the co-design project. At the protocol development stage, consultation was carried out with parents, school staff/governors, leading experts in universal screening and interventions in primary school settings, and representatives from key policy and practitioner

For peer review only - http://bmjopen.bmj.com/site/about/guidelines.xhtml

organisations. Examples of decisions that were made on the basis of this consultation include specifically focussing recruitment on children in Year 4 (age 8-9 years) on the basis that this would be a manageable time for primary schools, would allow primary schools to see the benefit, and for children to benefit when managing subsequent key transitions (e.g. to secondary school).

Throughout the co-design process we will consult with stakeholders in the following ways: (i) two parents with relevant lived experience, two school leaders and one school mental health lead for a charity are members of the study management group and will contribute to all decisions made at a strategic level; (ii) this dedicated stakeholder group will meet to review data and to make decisions to address how to solve problems and manage potentially conflicting points of view that have arisen through the co-design process; and (iii) an online PPI group will be formed to access wider views and will be invited to provide feedback on key issues that arise and need to be resolved throughout the co-design process.

Co-design Participants

Participants will include Y4 children (aged 8-9 years), parents of Y4 children, primary school staff and other stakeholders. Expected participant numbers for each group and at each stage in the co-design process are outlined in Table 1. These numbers are approximate and final numbers will be

For peer review only

informed by reviewing the range of perspectives represented in the sample and the information provided by participants.

To recruit participants with a broad range of perspectives to Stage 1 we will circulate study invitations to parents of all Year 4 children in two primary schools in the local Oxfordshire area, as well as using online on social media, and mailing lists to recruit parents with particular experiences. For the subsequent stages we will first contact school leaders to invite their school to participate, and circulate study information to Y4 parents and children inviting them to participate.

All adult participants will be required to give consent and children will be required to assent to participate in all stages of the project.

Inclusion criteria:

Children will be eligible to participate if they are in Y4 in a mainstream primary school in England, with parent/carer consent for their participation (Stages 1-4).

Parent/carers of children in Y4 in mainstream primary schools in England will be eligible to take part in Stages 1-4. However for Stage 1, we will also recruit parents through other routes in order to capture a range of experiences that might be particularly relevant to parents' engagement with and the accessibility of the pathway procedures, specifically parents who have a child with past/present mental health problem(s) or who is adopted/fostered, or where a parent has

For peer review only

past/present mental health problem(s), or is in the military (due to their experience of frequent relocations, extended separation from parents, and parental physical or psychological injuries [19]).

School staff will be included if they are employed in a mainstream primary/junior school in England (e.g. class teacher, headteacher) (Stages 1-4).

The inclusion criteria for wider stakeholders is that they must be a member of an organisation that is responsible for policy or practice relating to mental health provision in primary schools in England (e.g. commissioning group, local authority, mental health service provider, local policy maker organisation, or a governor in mainstream primary/junior schools) (Stage 1 and 4).

Table 1

Recruitment estimates for the co-design

Assessment	Planned N			
	Stakeholders	Teachers	Parents	Children
Stage 1. Initial focus groups	2	7	16	9
Stage 2. Administering procedures			144	144

For peer review only

Stage 3. Cued recall interviews		4	12	
Stage 4. Post-screening interviews/focus groups	12	12	12	12

Procedure

We will collect data at four stages to inform the development of the pathway (see Figure 1).

Stage 1. In this stage we will carry out in-depth one-to-one interviews and focus groups with stakeholders, school staff, children and parents. Focus groups and interviews will draw on questioning techniques informed by the Critical Incident Approach [20] to explore participants' views about features of the pathway which might help or hinder a positive experience, or which might have been overlooked by the pathway planners altogether. Focus groups and interviews will include table-top activities where participants are shown visual representations of different aspects of the pathway and they will be asked to discuss and write on provided notecards which will be placed on the table about their thoughts, feelings and concerns, with questions including: "What would be the best way to do this?", "Who do you think would be best placed to do

For peer review only

this?", "What might need to be done to help this part happening?","Where would be the best place for this to happen?","When is the best time to do this?", "Do you have any concerns about this part of the pathway?". Photographs will be taken of the visuals produced in the focus groups and interviews for analysis. Interviews and focus groups will be audio-recorded and transcribed verbatim. This data will be used to develop a detailed prototype set of procedures for screening, feedback and intervention delivery through schools to be tested and developed further. The dedicated stakeholder group will be consulted at key decision making points in the process to generate solutions to problems raised or inconsistent messages elicited from the interviews.

Stage 2. The detailed prototype set of procedures developed in Stage 1 will be administered in two primary schools, including screening, feedback to parents, and the offer of treatment where indicated. We will conduct interviews with school staff, children and parents (including children who screened 'negative' and those who screened 'positive' for anxiety and their parents, and parents that did and did not take up the intervention) to examine their experiences of the pathway and potential barriers/facilitators to engagement.

Stage 3. On the basis of the dedicated stakeholder input at the protocol design stage, we anticipate that parents will be given written feedback on their child's screening outcomes by a member of school staff, with the option of a face-to-face

For peer review only - http://bmjopen.bmj.com/site/about/guidelines.xhtml

feedback appointment. To understand how this feedback is experienced, what works well and what parents (and school staff) find both helpful and challenging, participating parents and staff will be invited to take part in a cued-recall interview meeting to allow for the refinement of future feedback delivery and staff training. To this effect, the parent-staff feedback meetings will be video recorded by the research team. Recordings of the meeting will be watched back by parents with a member of the research team to facilitate discussions about how questionnaire scores were fed back and how the opportunity to take up the intervention was shared with parents by school staff. The researcher will invite the parent to stop the recording periodically to comment at points that are relevant to particular cues, for example, at points where the parent felt the information delivered was unhelpful, or where they felt listened to. The cued-recall interview will be audio-recorded and transcribed verbatim.

Stage 4. Following the administration of the pathway procedures, interviews and focus groups will also be carried out with Y4 children, their parents and school staff in Stage 4. We will carry out interviews with participating parents and children who completed the screening questionnaires and engage with the treatment modules, but also with any parents and children who withdraw and parents and children who choose not to enroll in the study. School staff in participating schools will be interviewed about their experience of facilitating the

For peer review only - http://bmjopen.bmj.com/site/about/guidelines.xhtml

iCATS pathway in Y4. Relevant stakeholders will also be intereviewed about their views of the pathway and how well it will fit within school settings.

Data analysis

Focus groups and one-to-one interviews (Stages 1 and 4) will be analysed using two approaches: ‘fast and direct’ and ‘in-depth and detailed’. The ‘fast and direct’ analysis will use the visual outputs from focus groups and interviews to collate themes and written summaries will provide readily understandable feedback about the pathway. Brief, complementary descriptions will be produced by following a simple protocol for verbalising ‘multi- modal data’ [21]The combination of thematised images and verbal summary will provide immediate, easily- understood feedback about the pathway.

The ‘in-depth and detailed’ analysis will involve Template Analysis [22] where an initial template is structured by categories drawn from relevant literature and further developed by preliminary coding of the data using a ‘bottom up’ approach. Once the template is developed, all transcripts will be analysed in a ‘top down’ manner following the provisional structure of the template. This will provide nuanced feedback about the acceptability of the pathway to fine-tune the final iteration. This analysis will capture areas of disagreement that may be missed in the ‘fast and direct’ analysis. Cued-recall data (Stage 3) will also be analysed using Template Analysis [22]. Credibility will be checked via analytic triangulation using reflective discussions with co-analysts.

Ethics and dissemination

For peer review only - http://bmjopen.bmj.com/site/about/guidelines.xhtml

This research is being conducted in a community setting and ethical approval has been obtained from the University of Oxford's Research Ethics Committee. We will seek consent from parents, school staff and other stakeholders and assent from children. Research data will be kept secure and confidential. Audio/video-recordings will require explicit consent.

A key part of this project will involve developing acceptable procedures for feeding back outcomes from screening questionnaires which may bring potential to cause participant distress. Given that existing screening questionnaires have modest sensitivity and specificity, this includes explaining the possibility of an inaccurate result. We will pay particular attention to this throughout the co-design process to ensure we develop acceptable procedures and will seek out families who received 'false-positive' screening feedback to specifically explore their experience. Given the risk of 'false negatives', during this co-design phase the online intervention will be made available to all families who take part, along with information about additional resources, support and services.

This project aims to develop an effective pathway to identify child anxiety difficulties in mainstream schools and deliver a parent-led intervention through ongoing collaborative work with schools, parents, children and stakeholders, while fostering avenues for disseminating the results directly to the community. Specifically, this co-design study will lead on to a future feasibility study and,

For peer review only

if indicated, a randomised controlled trial. To disseminate the findings from this initial stage research, we will produce and disseminate a report that summarises outcomes in lay-language to participating schools, which will also be shared with participating families on request. The findings will be published in high-quality, open access journals that reach both academic, educational and clinical audiences. The research team will also present the findings at national and international clinical/educational conferences. We will also collaborate with our dedicated stakeholder group to further develop the dissemination plans to ensure maximum impact.

Discussion

There is currently no evidence-based pathway for identification and intervention for children with anxiety disorders in primary schools. Despite the existence of cost-effective psychological treatments, very few children who could benefit are able to access them [7]. This project aims to generate knowledge using a co-design approach to inform the development of such a pathway that links screening with the direct provision of support for primary school aged children to ensure that it is acceptable and ultimately implementable within schools in England. By providing information on children, parents, school-staff and other stakeholder's experiences of this school-based pathway which includes screening for likely child anxiety difficulties, feedback on scores and the offer of an online intervention, we anticipate

For peer review only - http://bmjopen.bmj.com/site/about/guidelines.xhtml

that the findings will also inform broader approaches to screening for and treating youth mental health problems outside of clinics.

This research has several methodological limitations that warrant consideration. First, because of the nature of child and parent involvement in this study, we will use an 'opt-in' approach to consent, where parents must consent to their and their child's completion of the screening measures. This is likely to introduce bias in participation, and we risk failing to capture experiences of the pathway procedures for a sufficiently broad and diverse group of families where child anxiety difficulties (including for example, families who do not have concerns about child anxiety or where other barriers may exist, such as concerns about stigma). To address this, we will actively invite parents to Stage 1 and 4 interviews who both did and did not consent to screening as well as examine in interviews whether an 'opt-out' approach to screening would be acceptable in future iterations. A second potential limitation is that the study will primarily use online platforms for consent, and screening procedures, and to deliver the CBT intervention. This decision was informed by feedback from the dedicated stakeholder group who recommended that online participation was often considered more secure in terms of data protection and privacy. However, it introduces a risk of excluding families who have limited access to technology/Wi-Fi or lack technical skills or confidence. We

For peer review only - http://bmjopen.bmj.com/site/about/guidelines.xhtml

will explore ways to enable participants in these situations to participate, and participant experiences of online access to the study will be examined.

With these potential limitations in mind, it is our intention that this study will collaboratively create a pathway to care for children who have problems with anxiety and their families, informed by children themselves, parents, school staff and other stakeholders, that will ultimately improve access to effective treatment and support.

References

1 Pfundmair M, Aßmann E, Kiver B, *et al.* Pathways toward Jihadism in Western Europe: An Empirical Exploration of a Comprehensive Model of Terrorist Radicalization. *Terror Polit Violence* 2019;:1–23. doi:10.1080/09546553.2019.1663828

2 Hofstra MB, Van Der Ende J, Verhulst FC. Continuity and change of psychopathology from childhood into adulthood: A 14-year follow-up study. *J Am Acad Child Adolesc Psychiatry* 2000;**39**:850–8. doi:10.1097/00004583-200007000-00013

3 Adams GC, Balbuena L, Meng XF, *et al.* When social anxiety and depression go together: A population study of comorbidity and associated consequences. *J Affect Disord* 2016;**206**:48–54. doi:10.1016/j.jad.2016.07.031

For peer review only - http://bmjopen.bmj.com/site/about/guidelines.xhtml

4 Brook JS, Zhang C, Rubenstone E, *et al.* Comorbid trajectories of substance use as predictors of Antisocial Personality Disorder, Major Depressive Episode, and Generalized Anxiety Disorder. *Addict Behav* 2016;**62**:114–21. doi:10.1016/j.addbeh.2016.06.003

5 Bodden DHM, Dirksen CD, Bögels SM. Societal burden of clinically anxious youth referred for treatment: A cost-of-illness study. *J Abnorm Child Psychol* 2008;**36**:487–97. doi:10.1007/s10802-007-9194-4

6 Essau CA, Muris P, Ederer EM. Reliability and validity of the Spence Children's Anxiety Scale and the Screen for Child Anxiety Related Emotional Disorders in German children. *J Behav Ther Exp Psychiatry* 2002;**33**:1–18. doi:10.1016/S0005-7916(02)00005-8

7 Reardon T, Harvey K, Creswell C. Seeking and accessing professional support for child anxiety in a community sample. *Eur Child Adolesc Psychiatry* 2020;**29**:649–64. doi:10.1007/s00787-019-01388-4

8 Rooksby M, Elouafkaoui P, Humphris G, *et al.* Internet-assisted delivery of cognitive behavioural therapy (CBT) for childhood anxiety: Systematic review and meta-analysis. J. Anxiety Disord. 2015;**29**:83–92. doi:10.1016/j.janxdis.2014.11.006

9 Fondo de las Naciones Unidas para la Infancia. Primary School Age Education. 2019. https://data.unicef.org/topic/education/primary-education/

For peer review only

(accessed 15 Sep 2020).

10 DIMOPOULOS-BICK, DAWDA P, MAHER L, *et al.* Experience-Based Co-Design: Tackling common challenges | The Journal of Health Design. *J Heal Des* 2019;**3**:86–93.https://www.journalofhealthdesign.com/JHD/article/view/46 (accessed 15 Sep 2020).

11 Bevan Jones R, Stallard P, Agha SS, *et al.* Practitioner review: Co-design of digital mental health technologies with children and young people. J. Child Psychol. Psychiatry Allied Discip. 2020;**61**:928–40. doi:10.1111/jcpp.13258

12 Palmer VJ, Chondros P, Piper D, *et al.* The CORE study protocol: A stepped wedge cluster randomised controlled trial to test a co-design technique to optimise psychosocial recovery outcomes for people affected by mental illness in the community mental health setting. *BMJ Open* 2015;**5**:e006688. doi:10.1136/bmjopen-2014-006688

13 Boyd H, McKernon S, Mullin B, *et al.* Improving healthcare through the use of co-design. *N Z Med J* 2012;**125**.https://www.nzma.org.nz/journal-articles/improving-healthcare-through-the-use-of-co-design (accessed 15 Sep 2020).

14 Reardon T, Spence SH, Hesse J, *et al.* Identifying children with anxiety disorders using brief versions of the Spence Children's Anxiety Scale for children, parents, and teachers. *Psychol Assess* 2018;**30**:1342–55.

For peer review only - http://bmjopen.bmj.com/site/about/guidelines.xhtml

doi:10.1037/pas0000570

15 D'Souza CM, Forman SF, Austin SB. Follow-up evaluation of a high school eating disorders screening program: Knowledge, awareness and self-referral. *J Adolesc Heal* 2005;**36**:208–13. doi:10.1016/j.jadohealth.2004.01.014

16 Thirlwall K, Cooper PJ, Karalus J, *et al.* Treatment of child anxiety disorders via guided parent-delivered cognitive-behavioural therapy: Randomised controlled trial. *Br J Psychiatry* 2013;**203**:436–44. doi:10.1192/bjp.bp.113.126698

17 Creswell C, Violato M, Fairbanks H, *et al.* Clinical outcomes and cost-effectiveness of brief guided parent-delivered cognitive behavioural therapy and solution-focused brief therapy for treatment of childhood anxiety disorders: a randomised controlled trial. *The Lancet Psychiatry* 2017;**4**:529–39. doi:10.1016/S2215-0366(17)30149-9

18 Thirlwall K, Cooper P, Creswell C. Guided parent-delivered cognitive behavioral therapy for childhood anxiety: Predictors of treatment response. *J Anxiety Disord* 2017;**45**:43–8. doi:10.1016/j.janxdis.2016.11.003

19 Williamson V, Stevelink SAM, Da Silva E, *et al.* A systematic review of wellbeing in children: a comparison of military and civilian families. *Child Adolesc Psychiatry Ment Health* 2018;**12**:46. doi:10.1186/s13034-018-0252-1

For peer review only - http://bmjopen.bmj.com/site/about/guidelines.xhtml

20 Butterfield LD, Borgen WA, Amundson NE, *et al.* Fifty years of the critical incident technique: 1954-2004 and beyond. *Qual Res* 2005;**5**:475–97. doi:10.1177/1468794105056924

21 Boden Z, Eatough V. Understanding More Fully: A Multimodal Hermeneutic-Phenomenological Approach. *Qual Res Psychol* 2014;**11**:160–77. doi:10.1080/14780887.2013.853854

22 Brooks J, McCluskey S, Turley E, *et al.* The Utility of Template Analysis in Qualitative Psychology Research. *Qual Res Psychol* 2015;**12**:202–22. doi:10.1080/14780887.2014.955224

For peer review only - http://bmjopen.bmj.com/site/about/guidelines.xhtml

BMJ Open

Protocol for the co-design and development of a primary school-based pathway for child anxiety screening and intervention delivery: A mixed-methods feasibility study

Journal:	*BMJ Open*
Manuscript ID	bmjopen-2020-044852.R1
Article Type:	Protocol
Date Submitted by the Author:	01-Feb-2021
Complete List of Authors:	Williamson, Victoria; King's College London; Oxford University<br>Larkin, Michael; Aston University<br>Reardon, Tessa; Oxford University<br>Pearcey, Samantha; Oxford University<br>Hill, Claire; University of Reading<br>Stallard, Paul; University of Bath<br>Spence, Susan; Griffith University<br>Breen, Maria; School of Psychology & Clinical Language Sciences NIHR ARC South West Peninsula, University of Reading, Reading, RG6 6ALExeter, Exeter, EX1 2LU<br>Macdonald, Ian; Charlie Waller Institute<br>Ukoumunne, Obioha; University of Exeter Medical School, NIHR CLAHRC South West Peninsula (PenCLAHRC)<br>Ford, Tamsin; University of Exeter; Cambridge University<br>Violato, Mara; Oxford University, Health Economics Research Centre, Nuffield Department of Population Health<br>Sniehotta, Falko; Newcastle University<br>Stainer, Jason; Stanley Primary School, Strathmore Road, Teddington, Middlesex, TW11 8UH<br>Gray, Alastair; Oxford University, Nuffield Department of Population Health<br>Brown, Paul; Bransgore C Of E Primary School, Ringwood Rd, Bransgore, Christchurch BH23 8JH<br>Sancho, Michelle; West Berkshire Council, Council Offices, Market St, Newbury RG14 5LD<br>Creswell , Cathy ; University of Oxford,
**Primary Subject Heading**:	Mental health
Secondary Subject Heading:	Evidence based practice, Paediatrics, Qualitative research
Keywords:	Anxiety disorders < PSYCHIATRY, Protocols & guidelines < HEALTH SERVICES ADMINISTRATION & MANAGEMENT, MENTAL HEALTH, Child & adolescent psychiatry < PSYCHIATRY, QUALITATIVE RESEARCH

SCHOLARONE™ Manuscripts

I, the Submitting Author has the right to grant and does grant on behalf of all authors of the Work (as defined in the below author licence), an exclusive licence and/or a non-exclusive licence for contributions from authors who are: i) UK Crown employees; ii) where BMJ has agreed a CC-BY licence shall apply, and/or iii) in accordance with the terms applicable for US Federal Government officers or employees acting as part of their official duties; on a worldwide, perpetual, irrevocable, royalty-free basis to BMJ Publishing Group Ltd ("BMJ") its licensees and where the relevant Journal is co-owned by BMJ to the co-owners of the Journal, to publish the Work in this journal and any other BMJ products and to exploit all rights, as set out in our licence.

The Submitting Author accepts and understands that any supply made under these terms is made by BMJ to the Submitting Author unless you are acting as an employee on behalf of your employer or a postgraduate student of an affiliated institution which is paying any applicable article publishing charge ("APC") for Open Access articles. Where the Submitting Author wishes to make the Work available on an Open Access basis (and intends to pay the relevant APC), the terms of reuse of such Open Access shall be governed by a Creative Commons licence – details of these licences and which Creative Commons licence will apply to this Work are set out in our licence referred to above.

Other than as permitted in any relevant BMJ Author's Self Archiving Policies, I confirm this Work has not been accepted for publication elsewhere, is not being considered for publication elsewhere and does not duplicate material already published. I confirm all authors consent to publication of this Work and authorise the granting of this licence.

For peer review only

Protocol for the co-design and development of a primary school-based pathway for child anxiety screening and intervention delivery: A mixed-methods feasibility study

Victoria, Williamson.[a,b*], Michael Larkin.[c] , Tessa Reardon[a] , Samantha Pearcey[a], Claire Hill[d], Paul Stallard[e], Sue Spence[f], Maria Breen[d], Ian Mcdonald[g], ObiohaUkoumunne[n,], Tamsin Ford[h], Mara Violato[i], Falko Sniehotta[j], Jason Stainer[k], Alastair Gray[i], Paul Brown[l], Michelle Sancho[m], Cathy Creswell. [a]

[a] Department of Experimental Psychology, Anna Watts Building, University of Oxford, Oxford, OX2 6 GG

[b]Institute of Psychiatry, Psychology and Neuroscience, King's College London, London, SE5 9RJ

[c] Aston Neuroscience Institute, Department of Psychology, Aston University, Birmingham, B4 7ET

[d] School of Psychology & Clinical Language Sciences, University of Reading, Reading, RG6 6AL

[e] University of Bath, Claverton Down, Bath BA2 7AY

[f] Griffith University, 16 Russell Street South Bank, QLD 4101, AU

[g] Charlie Waller Memorial Trust, First Floor, Rear Office, 32 High Street, Thatcham RG19 3JD

[h] Department of Psychiatry, University of Cambridge, Cambridge, UK

For peer review only

[I] Nuffield Department of Population Health, University of Oxford, Richard Doll Building, Old Road Campus, Oxford OX3 7LF | UK

[j] Population Health Science Institute, Faculty of Medical Sciences, Newcastle University, Baddiley-Clark Building, Richardson Road, NE2 4AX

[k] Stanley Primary School, Strathmore Road, Teddington, Middlesex, TW11 8UH

[l] Bransgore C Of E Primary School, Ringwood Rd, Bransgore, Christchurch BH23 8JH

[m] West Berkshire Council, Council Offices, Market St, Newbury RG14 5LD

[n] NIHR ARC South West Peninsula, University of Exeter, Stocker Rd, Exeter EX4 4PY

***Correspondence:** Dr Victoria Williamson, Department of Experimental Psychology, University of Oxford, Oxford, OX2 6 GG; Institute of Psychiatry, Psychology and Neuroscience, King's College London, London, SE5 9RJ. Victoria.williamson@kcl.ac.uk

Word Count: 3944

For peer review only

Abstract

Introduction: Anxiety difficulties are among the most common mental health problems in childhood. Despite this, few children access evidence-based interventions and school may be an ideal setting to improve children's access to treatment. This paper describes the design, sampling methods, and expected data collection of the Identifying Child Anxiety Though Schools – Identification to Intervention (iCATS i2i) study which aims to develop acceptable school-based procedures to identify and support child anxiety difficulties.

Methods and analysis: iCATS i2i will use a mixed-methods approach to co-design and deliver a set of procedures – or 'pathway' – to improve access to evidence-based intervention for child anxiety difficulties through primary schools in England. The study will consist of four stages, initially involving in-depth interviews with parents, children, school staff, stakeholders (Stage 1) to inform the development of the pathway. The pathway will then be administered in two primary schools, including screening, feedback to parents, and the offer of treatment where indicated (Stage 2), with participating children, parents and school staff invited to provide feedback on their experience (Stages 3 & 4). Data will be analysed using Template Analysis.

Ethics and dissemination: The iCATS i2i study was approved by the University of Oxford's Research Ethics Committee (REF R64620/RE001). It is expected that this co-design study will lead on to a future

For peer review only - http://bmjopen.bmj.com/site/about/guidelines.xhtml

For peer review only

feasibility study and, if indicated, a randomised controlled trial. By providing information on child, parent, school-staff and other stakeholder's experiences, we anticipate that the findings will inform the development of an acceptable evidence-based pathway for identification and intervention for children with anxiety difficulties in primary schools and may also inform broader approaches to screening for and treating youth mental health problems outside of clinics.

Strengths and limitations of this study:

- Focus on child anxiety difficulties, one of the most common mental helath problems in childhood.
- By using a co-design approach which incorporates feedback from children, parents, school-staff and stakeholders, this study will lead to the development of acceptable procedures for screening and offering treatment for child anxiety difficulties in primary schools.
- The study is limited by the use of an 'opt-in' approach to consent which could introduce participation bias.
- The primary use of online platforms for consent, screening and delivery of the CBT intervention may exclude families who have limited access to technology or lack technical skills; although ways to facilitate the participation of those in these situations will be explored.

For peer review only

For peer review only

For peer review only - http://bmjopen.bmj.com/site/about/guidelines.xhtml

Introduction

Anxiety difficulties are among the most common mental health problems in childhood (6.5% prevalence; [1]) and approximately half of all anxiety difficulties emerge by the age of 11 years. Childhood anxiety difficulties are often chronic and pervasive, and have an adverse effect on social, education and familial functioning. Childhood anxiety difficulties often persist into adulthood when left untreated [2] and are associated with comorbid mental health difficulties, including major depression and substance abuse [3,4]. The societal cost of a child with an anxiety difficulty is estimated to be 21 times that of a non-anxious child [5]. As such, effective identification and treatment of anxiety difficulties in childhood is important.

Effective treatments for childhood anxiety exist. However, very few children are offered or are able to access them [6,7]. For example, previous research has shown that only 2% of pre-adolescent children who meet criteria for an anxiety disorder in England receive an evidence-based intervention [7]. Barriers to receiving evidence-based treatment can include problems with the identification of anxiety difficulties, concerns regarding stigma to the child or family, as well as a scarcity of trained mental health professionals and long waiting lists for specialist services [8,9]. Practically speaking, attending group or face-to-face programs can also bring logistical barriers for parents with

For peer review only - http://bmjopen.bmj.com/site/about/guidelines.xhtml

young families including time demands, and difficulties with arranging transportation or child care [10-12].

The vast majority of children attend and spend much of their time at school, therefore schools are also an ideal setting to overcome many of these barriers [12,13]. However, there is not a clear set of procedures for identifying youth mental health difficulties and promoting access to evidence-based treatments in schools. Moreover, previous international studies have found mixed support for school based screening and interventions for childhood anxiety, with some studies reporting reductions in child anxiety symptoms [12,14], while other studies have not [15]. Furthermore, some studies have reported low uptake to school-based interventions, for reasons including parents finding screening questionnaires too time consuming, parent concerns about stigma, as well as fears that their child may become more anxious from having had to discuss their worries [14]. This highlights the need for novel approaches to promote school based approaches to increase access to early intervention for childhood anxiety difficulties that are acceptable and well tolerated in order to to increase parent participation.

One approach often used in healthcare service design and development is 'co-design', where the knowledge and lived experiences of service users themselves are drawn upon to enhance the quality and experiences of care [16,17]. Co-design aims to develop an in-depth understanding of how stakeholders

For peer review only - http://bmjopen.bmj.com/site/about/guidelines.xhtml

and service users perceive and experience the look, feel, procedures and structures of a service [18]. By engaging stakeholders and service users in co-designing a service, this is thought to lead to better quality of care and improved service performance by highlighting individual's subjective experiences at various points in the care pathway, which, in turn, may lead to improvements in health outcomes and more efficient allocation of limited healthcare resources [19]. Given the importance of early intervention, an acceptable school-based pathway that incorporates the identification of children with anxiety difficulties and promotes uptake of evidence-based intervention is urgently needed. The Identifying Child Anxiety Though Schools – Identification to Intervention (iCATS i2i) study will develop procedures to identify and support child anxiety difficulties through schools informed by a co-design approach.

This article describes the iCATS i2i co-design protocol. Data collection for this study will take place between December 2019 - December 2020. The co-designed procedures will be evaluated in a subsequent feasibility study and, if indicated, randomised control trial beginning in 2021.

Method

This protocol and associated procedures were approved by the Central University Research Ethics Committee at the University of Oxford (REF R64620/RE001).

Study design

For peer review only - http://bmjopen.bmj.com/site/about/guidelines.xhtml

We will apply a mixed-methods approach to co-design, produce and deliver a set of procedures – or 'pathway' - to improve access to evidence-based intervention for child anxiety difficulties through primary schools (i.e. ages 5-11) in England. Several of the key elements of the pathway were specified in advance of the co-design work based on the existing empirical literature and parent and school staff consultation. Specifically, it was pre-specified that children's anxiety difficulties would be screened using questionnaire measures, parents would receive feedback, and, where indicated, a brief, parent-led online intervention for child anxiety difficulties would be offered. The delivery of online treatment directly to parents was included as it has potential to overcome many of the barriers to care described above, such as logistical issues for parents and parental concerns about negative impacts on the child of participating in treatment [7,8].

In parallel to this work we are working on refining measures for screening for child anxiety problems (Reardon et al. under review), but in the interim we will screen using brief child, parent and teacher versions of the Spence Child Anxiety Scale (SCAS-8;[20]) together with four items that assess the extent of interference in everyday life (e.g. "Do fears and worries stop you from doing things?") generated to assess the impact and chronicity of and perceived need for help for anxiety difficulties. The addition of interference

For peer review only - http://bmjopen.bmj.com/site/about/guidelines.xhtml

items is known to improve the efficacy of similar self-report measures [21]. We will consider a child to have screened ‘positive’ for likely anxiety difficulties if they score above the cut off on the SCAS-8 on the basis of any reporter (score of 7.5 for parents, 6.5 for children, 4.5 for teachers) and/or indicate that anxiety interferes at least 1 ‘only a little’ on any of the interferance items. This interference-based cut off score was based on feedback from the dedicated stakeholder group. The use of a screening questionnaire to determine which children may benefit from additional support with anxiety was a prespecified component of the study as this approach shows promise for increasing access to support (e.g. [22]).

The intervention to be offered is an online version of a brief therapist-guided parent-delivered CBT approach for child anxiety difficulties (OSI; Online Support and Intervention for child anxiety). OSI was originally developed for use in National Health Service (NHS) clinics and was co-designed by NHS clinicians, parents and children who had received treatment for anxiety (Hill et al., in preparation). This intervention was selected as it is brief, effective [23] and more cost-effective than brief face-to-face psychological therapy [24] and can be delivered by non-expert practitioners (e.g. [25]). The approach of working directly with the parent, rather than the child, also addressed particular barriers to seeking and accessing help for anxiety highlighted by parents, including the preference to be supported to manage the

For peer review only - http://bmjopen.bmj.com/site/about/guidelines.xhtml

difficulties as a family and for the child not to be singled out [7]. The online version of this intervention involves 7 online modules for parents, supported by a weekly 20-minute telephone call with a Children's Wellbeing Practitioner (CWP, NHS Band 5), with a follow-up telephone session 4-weeks later. Modules teach parents how to explore their child's anxious thoughts, put them to the test through facing fears, and to problem solve challenges that arise. This is accompanied by a game app for the child to help motivate them to face their fears. The CWPs are postgraduate psychological therapists who have received specific (12 month) training in the delivery of brief psychological therapies for children and young people who have difficulties with anxiety, low mood, and behavioural disturbance. CWPs are based within settings where they can offer rapid access to psychological therapies, often including school based clinical services, and so are the ideal workforce to implement the approach being developed if indicated.

We will use a mixed-method co-design process to determine *how* the pre-specified parts of the pathway should be presented, who by, and to address any important considerations to optimise accessibility of and engagement with the pathway. The co-design process will consist of four stages (see Figure 1) involving initial interviews and focus groups with parents, children, school staff, stakeholders (Stage 1) to inform the development of a set of procedures that will be applied in two schools. These procedures will be delivered with participating

For peer review only - http://bmjopen.bmj.com/site/about/guidelines.xhtml

children, parents and school staff (Stage 2) who will provide feedback on their experience (Stage 3 & 4), including cued-recall specifically on the experience of receiving feedback on whether their child experiences difficulties with anxiety. Feedback from those families who choose not to be involved in the study or dropped out will also be sought to ensure any barriers to engagement are captured (Stage 4).

.

Patient and public involvement (PPI)

Involvement from parents, school staff and wider stakeholders informed the development of this protocol, the pre-specified elements of the pathway and will contribute throughout the delivery of the co-design project. At the protocol development stage, consultation was carried out with parents, school staff/governors, leading experts in universal screening and interventions in primary school settings, and representatives from key policy and practitioner organisations. Examples of decisions that were made on the basis of this consultation include specifically focussing recruitment on children in Year 4 (age 8-9 years) on the basis that this would be a manageable time for primary schools, would allow primary schools to see the benefit, and for children to benefit when managing subsequent key transitions (e.g. to secondary school).

For peer review only - http://bmjopen.bmj.com/site/about/guidelines.xhtml

Throughout the co-design process we will consult with stakeholders in the following ways: (i) two parents with relevant lived experience, two school leaders and one mental health lead for a charity are members of the study management group and will contribute to all decisions made at a strategic level; (ii) this dedicated stakeholder group will meet to review data and to make decisions to address how to solve problems and manage potentially conflicting points of view that have arisen through the co-design process; and (iii) a distinct, separate online PPI group will also be formed, made up primarily of parents. Members will be invited to join via the circulation of adverts about the online group (e.g. advert shared on social media, circulation of advert to parents from participating Stage 2 schools), with the purpose of the group being to access wider parental views about study procedures and on key issues that arise during the study.

Co-design Participants

Participants will include Y4 children (aged 8-9 years), parents of Y4 children, primary school staff and other stakeholders. Expected participant numbers for each group and at each stage in the co-design process are outlined in Table 1. These numbers are approximate and final numbers will be informed by reviewing the range of perspectives represented in the sample and the information provided by participants.

To recruit participants with a broad range of perspectives to Stage 1 we will circulate study invitations to

For peer review only - http://bmjopen.bmj.com/site/about/guidelines.xhtml

parents of all Year 4 children in two primary schools in the local Oxfordshire area, as well as using online on social media, and mailing lists to recruit parents with particular experiences. For the subsequent stages we will first contact school leaders to invite their school to participate, and circulate study information to Y4 parents and children inviting them to participate.

All adult participants will be required to give written consent and children will be required to give written assent to participate in all stages of the project.

Inclusion criteria:

Children will be eligible to participate if they are in Y4 in a mainstream primary school in England, with parent/carer consent for their participation (Stages 1-4).

Parent/carers of children in Y4 in mainstream primary schools in England will be eligible to take part in Stages 1-4. However for Stage 1, we will also recruit parents through other routes in order to capture a range of experiences that might be particularly relevant to parents' engagement with and the accessibility of the pathway procedures, specifically parents who have a child with past/present mental health problem(s) or who is adopted/fostered, or where a parent has past/present mental health problem(s), or is in the military (due to their experience of frequent relocations, extended separation from parents, and parental physical or psychological injuries [26]).

For peer review only - http://bmjopen.bmj.com/site/about/guidelines.xhtml

School staff will be included if they are employed in a mainstream primary/junior school in England (e.g. class teacher, headteacher) (Stages 1-4).

The inclusion criteria for wider stakeholders is that they must be a member of an organisation that is responsible for policy or practice relating to mental health provision in primary schools in England (e.g. commissioning group, local authority, mental health service provider, local policy maker organisation, or a governor in mainstream primary/junior schools) (Stage 1 and 4).

Table 1

Recruitment estimates for the co-design

Assessment	Planned N			
	Stakeholders	Teachers	Parents	Children
Stage 1. Initial focus groups	2	7	16	9
Stage 2. Administering procedures			144	144
Stage 3. Cued recall interviews		4	12	
Stage 4. Post-screening	12	12	12	12

For peer review only - http://bmjopen.bmj.com/site/about/guidelines.xhtml

interviews/focus groups

Procedure

We will collect data at four stages to inform the development of the pathway (see Figure 1).

Stage 1. In this stage we will carry out in-depth one-to-one interviews and focus groups with stakeholders, school staff, children and parents. Focus groups and interviews will draw on questioning techniques informed by the Critical Incident Approach [27] to explore participants' views about features of the pathway which might help or hinder a positive experience, or which might have been overlooked by the pathway planners altogether. Focus groups and interviews will include table-top activities where participants are shown visual representations of different aspects of the pathway and they will be asked to discuss and write on provided notecards which will be placed on the table about their thoughts, feelings and concerns, with questions including: "What would be the best way to do this?", "Who do you think would be best placed to do this?", "What might need to be done to help this part happening?","Where would be the best place for this to happen?","When is the best time to do this?", "Do you have any concerns about this part of the pathway?". Photographs will be taken of the visuals produced in the focus groups and

For peer review only - http://bmjopen.bmj.com/site/about/guidelines.xhtml

interviews for analysis. Interviews and focus groups will be audio-recorded and transcribed verbatim. This data will be used to develop a detailed prototype set of procedures for screening, feedback and intervention delivery through schools to be tested and developed further. The dedicated stakeholder group will be consulted at key decision making points in the process to generate solutions to problems raised or inconsistent messages elicited from the interviews.

Stage 2. The detailed prototype set of procedures developed in Stage 1 will be administered in two primary schools, including screening, feedback to parents, and the offer of early intervention where indicated. We will encourage each school to nominate a member of staff to be the 'pathway lead' (e.g. a class teacher, the school's pastoral lead) who will be given training and psychoeducation by the research team about childhood anxiety difficulties and the proposed pathway procedures. The 'pathway lead' will coordinate recruitment efforts at their participating school, such as circulating study information sheets amongst Y4 parents. During Stage 2, we will quantitatively examine pathway outcomes, including the proportion of parents in Y4 who agree to participate in the screening, the number of eligible parents who take up the intervention, the number of parents who withdraw, and symptom improvement rates. We will also conduct interviews with school staff, children and parents (including children who screened 'negative' and those who

For peer review only - http://bmjopen.bmj.com/site/about/guidelines.xhtml

screened 'positive' for anxiety and their parents, and parents that did and did not take up the intervention) to examine their experiences of the pathway and potential barriers/facilitators to engagement.

Stage 3. On the basis of the dedicated stakeholder input at the protocol design stage, we anticipate that parents will be given written feedback on their child's screening outcomes by the school 'pathway lead', with the option of a face-to-face feedback appointment. The dedicated stakeholder group considered that feedback from the school 'pathway lead' would be preferred by families as families would likely have pre-existing relationships with the school and a member of school staff would therefore be well placed to introduce the CWP and the option to access the intervention. If this is supported by the outcomes of the earlier stages, the school staff member that is nominated to be the 'pathway lead' will receive training and guidance from the research team on delivering feedback to parents. To understand how this feedback is experienced, what works well and what parents (and the school staff 'pathway lead') find both helpful and challenging, participating parents and staff will be invited to take part in a cued-recall interview meeting to allow for the refinement of future feedback delivery and staff training. To this effect, the parent-staff feedback meetings will be video recorded by the research team. Recordings of the meeting will be watched back by parents with a member of the research team

For peer review only - http://bmjopen.bmj.com/site/about/guidelines.xhtml

to facilitate discussions about how questionnaire scores were fed back and how the opportunity to take up the intervention was shared with parents by 'pathway lead' school staff member. The researcher will invite the parent to stop the recording periodically to comment at points that are relevant to particular cues, for example, at points where the parent felt the information delivered was unhelpful, or where they felt listened to. The cued-recall interview will be audio-recorded and transcribed verbatim.

Stage 4. Following the administration of the pathway procedures, interviews and focus groups will also be carried out with Y4 children, their parents and school staff in Stage 4. We will carry out interviews with participating parents and children who completed the screening questionnaires and engage with the intervention modules, but also with any parents and children who withdraw and parents and children who choose not to enrol in the study. School staff (e.g., the 'pathway lead', Y4 class teachers) in participating schools will be interviewed about their experience of facilitating the pathway in Y4. Relevant stakeholders will also be interviewed about their views of the pathway and how well it will fit within school settings.

Data analysis

Focus groups and one-to-one interviews (Stages 1 and 4) will be analysed using two approaches: 'fast and direct' and 'in-depth and detailed'. The 'fast and direct' analysis will use the visual outputs from focus groups and interviews to collate themes and written

For peer review only - http://bmjopen.bmj.com/site/about/guidelines.xhtml

summaries will provide readily understandable feedback about the pathway. Brief, complementary descriptions will be produced by following a simple protocol for verbalising 'multi- modal data' [28]The combination of thematised images and verbal summary will provide immediate, easily- understood feedback about the pathway.

The 'in-depth and detailed' analysis will involve Template Analysis [29] where an initial template is structured by categories drawn from relevant literature and further developed by preliminary coding of the data using a 'bottom up' approach. Once the template is developed, all transcripts will be analysed in a 'top down' manner following the provisional structure of the template. This will provide nuanced feedback about the acceptability of the pathway to fine-tune the final iteration. This analysis will capture areas of disagreement that may be missed in the 'fast and direct' analysis. Cued-recall data (Stage 3) will also be analysed using Template Analysis [29]. Credibility will be checked via analytic triangulation using reflective discussions with co-analysts.

Ethics and dissemination

This research is being conducted in a community setting and ethical approval has been obtained from the University of Oxford's Research Ethics Committee. We will seek consent from parents, school staff and other stakeholders and assent from children. Research data will be kept secure and confidential. Audio/video-recordings will require explicit consent.

A key part of this project will involve developing acceptable procedures for feeding back outcomes from screening

For peer review only - http://bmjopen.bmj.com/site/about/guidelines.xhtml

questionnaires which may bring potential to cause participant distress. Given that existing screening questionnaires have modest sensitivity and specificity, this includes explaining the possibility of an inaccurate result. We will pay particular attention to this throughout the co-design process to ensure we develop acceptable procedures and will seek out families who received 'false-positive' screening feedback to specifically explore their experience. Given the risk of 'false negatives', during this co-design phase the online intervention will be made available to all families who take part, along with information about additional resources, support and services.

Dissemination. This project aims to develop an effective pathway to identify child anxiety difficulties in mainstream schools and deliver a parent-led intervention through ongoing collaborative work with schools, parents, children and stakeholders, while fostering avenues for disseminating the results directly to the community. Specifically, this co-design study will lead on to a future feasibility study and, if indicated, a randomised controlled trial. To disseminate the findings from this initial stage research, we will produce and disseminate a report that summarises outcomes in lay-language to participating schools, which will also be shared with participating families on request. The findings will be published in high-quality, open access journals that reach both academic, educational and clinical audiences. The

For peer review only - http://bmjopen.bmj.com/site/about/guidelines.xhtml

research team will also present the findings at national and international clinical/educational conferences. We will also collaborate with our dedicated stakeholder group to further develop the dissemination plans to ensure maximum impact.

Discussion

There is currently no evidence-based pathway for identification and intervention for children with anxiety difficulties in primary schools. Despite the existence of cost-effective psychological treatments, very few children who could benefit are able to access them [7]. This project aims to generate knowledge using a co-design approach to inform the development of such a pathway that links screening with the direct provision of support for primary school aged children to ensure that it is acceptable and ultimately implementable within schools in England. By providing information on children, parents, school-staff and other stakeholder's experiences of this school-based pathway which includes screening for likely child anxiety difficulties, feedback on scores and the offer of an online intervention, we anticipate that the findings will also inform broader approaches to screening for and treating youth mental health problems outside of clinics.

This research has several methodological limitations that warrant consideration. First, because of the nature of child and parent involvement in this study, we will use an 'opt-in' approach to consent, where parents must consent to their and

For peer review only

their child's completion of the screening measures. This is likely to introduce bias in participation, and we risk failing to capture experiences of the pathway procedures for a sufficiently broad and diverse group of families where child anxiety difficulties (including for example, families who do not have concerns about child anxiety or where other barriers may exist, such as concerns about stigma). To address this, we will actively invite parents to Stage 1 and 4 interviews who both did and did not consent to screening as well as examine in interviews whether an 'opt-out' approach to screening would be acceptable in future iterations (e.g., screening measures are administered to the entire Y4 class unless parents opt-out their child from participating). A second potential limitation is that the study will primarily use online platforms for consent, and screening procedures, and to deliver the CBT intervention. This decision was informed by feedback from the dedicated stakeholder group who recommended that online participation was often considered more secure in terms of data protection and privacy. However, it introduces a risk of excluding families who have limited access to technology/Wi-Fi or lack technical skills or confidence. We will explore ways to enable participants in these situations to participate, and participant experiences of online access to the study will be examined.

With these potential limitations in mind, it is our intention that this study will collaboratively create a

For peer review only

pathway to care for children who have problems with anxiety and their families, informed by children themselves, parents, school staff and other stakeholders, that will ultimately improve access to effective treatment and support.

Funding: This paper represents independent research funded by the National Institute for Health Research (NIHR; RP-PG-0218-20010) (CC). The views expressed are those of the authors and not necessarily those of the NHS, the NIHR or the Department of Health and Social Care.

Competing interests: Authors have no conflicts of interest to declare.

Contributorship statement: All authors (Victoria, Williamson.[a,b*], Michael Larkin.[c] , Tessa Reardon[a] , Samantha Pearcey[a], Claire Hill[d], Paul Stallard[e], Sue Spence[f], Maria Breen[d], Ian Mcdonald[g], ObiohaUkoumunne[n'], Tamsin Ford[h], Mara Violato[i], Falko Sniehotta[j], Jason Stainer[k], Alastair Gray[i], Paul Brown[l], Michelle Sancho[m], Cathy Creswell. [a]) contributed towards the design of the study protocol, contributed towards the writing of the manuscript and approved the final version of the protocol. Authors (Victoria, Williamson.[a,b*], Michael Larkin.[c] , Tessa Reardon[a] , Samantha Pearcey[a], Sue Spence[f], Ian Mcdonald[g], Tamsin Ford[h], Jason Stainer[k], Paul Brown[l], Cathy Creswell. [a]) contributed towards data collection and analysis.

For peer review only

For peer review only

For peer review only - http://bmjopen.bmj.com/site/about/guidelines.xhtml

References

1 Polanczyk G V., Salum GA, Sugaya LS, *et al.* Annual research review: A meta-analysis of the worldwide prevalence of mental disorders in children and adolescents. *J Child Psychol Psychiatry Allied Discip* 2015;**56**:345–65. doi:10.1111/jcpp.12381

2 Hofstra MB, Van Der Ende J, Verhulst FC. Continuity and change of psychopathology from childhood into adulthood: A 14-year follow-up study. *J Am Acad Child Adolesc Psychiatry* 2000;**39**:850–8. doi:10.1097/00004583-200007000-00013

3 Adams GC, Balbuena L, Meng XF, *et al.* When social anxiety and depression go together: A population study of comorbidity and associated consequences. *J Affect Disord* 2016;**206**:48–54. doi:10.1016/j.jad.2016.07.031

4 Brook JS, Zhang C, Rubenstone E, *et al.* Comorbid trajectories of substance use as predictors of Antisocial Personality Disorder, Major Depressive Episode, and Generalized Anxiety Disorder. *Addict Behav* 2016;**62**:114–21. doi:10.1016/j.addbeh.2016.06.003

5 Bodden DHM, Dirksen CD, Bögels SM. Societal burden of clinically anxious youth referred for treatment: A cost-of-illness study. *J Abnorm Child Psychol* 2008;**36**:487–97. doi:10.1007/s10802-007-9194-4

6 Essau CA, Muris P, Ederer EM. Reliability and validity of the Spence Children's Anxiety Scale and the Screen for

For peer review only - http://bmjopen.bmj.com/site/about/guidelines.xhtml

Child Anxiety Related Emotional Disorders in German children. *J Behav Ther Exp Psychiatry* 2002;**33**:1–18. doi:10.1016/S0005-7916(02)00005-8

7 Reardon T, Harvey K, Creswell C. Seeking and accessing professional support for child anxiety in a community sample. *Eur Child Adolesc Psychiatry* 2020;**29**:649–64. doi:10.1007/s00787-019-01388-4

8 Mojtabai R, Olfson M, Sampson NA, *et al.* Barriers to mental health treatment: results from the National Comorbidity Survey Replication. *Psychol Med* 2011;**41**. doi:10.1017/S0033291710002291

9 Schnyder N, Sawyer MG, Lawrence D, *et al.* Barriers to mental health care for Australian children and adolescents in 1998 and 2013–2014. *Aust N Z J Psychiatry* 2020;**54**. doi:10.1177/0004867420919158

10 Axford N, Lehtonen M, Kaoukji D, *et al.* Engaging parents in parenting programs: Lessons from research and practice. *Child Youth Serv Rev* 2012;**34**:2061–71. doi:10.1016/j.childyouth.2012.06.011

11 Morgan AJ, Rapee RM, Salim A, *et al.* Internet-Delivered Parenting Program for Prevention and Early Intervention of Anxiety Problems in Young Children: Randomized Controlled Trial. *J Am Acad Child Adolesc Psychiatry* 2017;**56**:417-425.e1. doi:10.1016/j.jaac.2017.02.010

12 Mifsud C, Rapee RM. Early intervention for childhood anxiety in a school setting: Outcomes for an economically

For peer review only - http://bmjopen.bmj.com/site/about/guidelines.xhtml

For peer review only

disadvantaged population. *J Am Acad Child Adolesc Psychiatry* 2005;**44**:996–1004. doi:10.1097/01.chi.0000173294.13441.87

13 Neil AL, Christensen H. Efficacy and effectiveness of school-based prevention and early intervention programs for anxiety. Clin. Psychol. Rev. 2009;**29**:208–15. doi:10.1016/j.cpr.2009.01.002

14 McLoone JK, Rapee RM. Comparison of an Anxiety Management Program for Children Implemented at Home and School: Lessons Learned. *School Ment Health* 2012;**4**:231–42. doi:10.1007/s12310-012-9088-7

15 Miller LD, Laye-Gindhu A, Liu Y, *et al.* Evaluation of a preventive intervention for child anxiety in two randomized attention-control school trials. *Behav Res Ther* 2011;**49**:315–23. doi:10.1016/j.brat.2011.02.006

16 Dimopoulos-Bick, Dawda P, Maher L, *et al.* Experience-Based Co-Design: Tackling common challenges | The Journal of Health Design. *J Heal Des* 2019;**3**:86–93.https://www.journalofhealthdesign.com/JHD/article/view/46 (accessed 15 Sep 2020).

17 Bevan Jones R, Stallard P, Agha SS, *et al.* Practitioner review: Co-design of digital mental health technologies with children and young people. J. Child Psychol. Psychiatry Allied Discip. 2020;**61**:928–40. doi:10.1111/jcpp.13258

18 Palmer VJ, Chondros P, Piper D, *et al.* The CORE study

For peer review only

protocol: A stepped wedge cluster randomised controlled trial to test a co-design technique to optimise psychosocial recovery outcomes for people affected by mental illness in the community mental health setting. *BMJ Open* 2015;**5**:e006688. doi:10.1136/bmjopen-2014-006688

19 Boyd H, McKernon S, Mullin B, *et al.* Improving healthcare through the use of co-design. *N Z Med J* 2012;**125**.https://www.nzma.org.nz/journal-articles/improving-healthcare-through-the-use-of-co-design (accessed 15 Sep 2020).

20 Reardon T, Spence SH, Hesse J, *et al.* Identifying children with anxiety disorders using brief versions of the Spence Children's Anxiety Scale for children, parents, and teachers. *Psychol Assess* 2018;**30**:1342–55. doi:10.1037/pas0000570

21 Evans R, Thirlwall K, Cooper P, *et al.* Using symptom and interference questionnaires to identify recovery among children with anxiety disorders. *Psychol Assess* 2017;**29**:835–43. doi:10.1037/pas0000375

22 D'Souza CM, Forman SF, Austin SB. Follow-up evaluation of a high school eating disorders screening program: Knowledge, awareness and self-referral. *J Adolesc Heal* 2005;**36**:208–13. doi:10.1016/j.jadohealth.2004.01.014

23 Thirlwall K, Cooper PJ, Karalus J, *et al.* Treatment of child anxiety disorders via guided parent-delivered cognitive-behavioural therapy: Randomised controlled

For peer review only - http://bmjopen.bmj.com/site/about/guidelines.xhtml

trial. *Br J Psychiatry* 2013;**203**:436–44. doi:10.1192/bjp.bp.113.126698

24 Creswell C, Violato M, Fairbanks H, *et al.* Clinical outcomes and cost-effectiveness of brief guided parent-delivered cognitive behavioural therapy and solution-focused brief therapy for treatment of childhood anxiety disorders: a randomised controlled trial. *The Lancet Psychiatry* 2017;**4**:529–39. doi:10.1016/S2215-0366(17)30149-9

25 Thirlwall K, Cooper P, Creswell C. Guided parent-delivered cognitive behavioral therapy for childhood anxiety: Predictors of treatment response. *J Anxiety Disord* 2017;**45**:43–8. doi:10.1016/j.janxdis.2016.11.003

26 Williamson V, Stevelink SAM, Da Silva E, *et al.* A systematic review of wellbeing in children: a comparison of military and civilian families. *Child Adolesc Psychiatry Ment Health* 2018;**12**:46. doi:10.1186/s13034-018-0252-1

27 Butterfield LD, Borgen WA, Amundson NE, *et al.* Fifty years of the critical incident technique: 1954-2004 and beyond. *Qual Res* 2005;**5**:475–97. doi:10.1177/1468794105056924

28 Boden Z, Eatough V. Understanding More Fully: A Multimodal Hermeneutic-Phenomenological Approach. *Qual Res Psychol* 2014;**11**:160–77. doi:10.1080/14780887.2013.853854

29 Brooks J, McCluskey S, Turley E, *et al.* The Utility of Template Analysis in Qualitative Psychology Research. *Qual*

For peer review only - http://bmjopen.bmj.com/site/about/guidelines.xhtml

Res Psychol 2015;**12**:202–22.

doi:10.1080/14780887.2014.955224

For peer review only

Figure 1. Overview of the co-design process for developing the iCATS i2i protocol

Note. Y4 = year four.

For peer review only

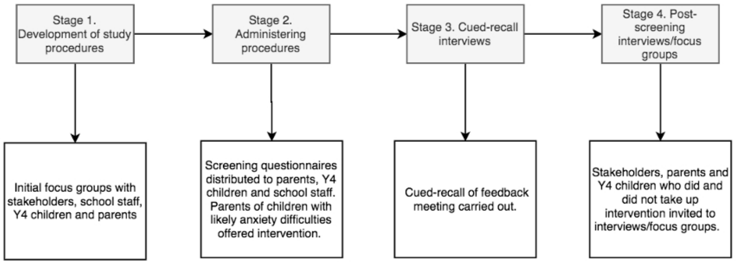

Figure 1. Overview of the co-design process for developing the iCATS i2i protocol

Note. Y4 = year four.

Figure 1

BMJ Open

Protocol for the co-design and development of a primary school-based pathway for child anxiety screening and intervention delivery: A mixed-methods feasibility study

Journal:	*BMJ Open*
Manuscript ID	bmjopen-2020-044852.R2
Article Type:	Protocol
Date Submitted by the Author:	03-Mar-2021
Complete List of Authors:	Williamson, Victoria; King's College London; Oxford University<br>Larkin, Michael; Aston University<br>Reardon, Tessa; Oxford University<br>Pearcey, Samantha; Oxford University<br>Hill, Claire; University of Reading<br>Stallard, Paul; University of Bath<br>Spence, Susan; Griffith University<br>Breen, Maria; School of Psychology & Clinical Language Sciences NIHR ARC South West Peninsula, University of Reading, Reading, RG6 6ALExeter, Exeter, EX1 2LU<br>Macdonald, Ian; Charlie Waller Institute<br>Ukoumunne, Obioha; University of Exeter Medical School, NIHR CLAHRC South West Peninsula (PenCLAHRC)<br>Ford, Tamsin; University of Exeter; Cambridge University<br>Violato, Mara; Oxford University, Health Economics Research Centre, Nuffield Department of Population Health<br>Sniehotta, Falko; Newcastle University<br>Stainer, Jason; Stanley Primary School, Strathmore Road, Teddington, Middlesex, TW11 8UH<br>Gray, Alastair; Oxford University, Nuffield Department of Population Health<br>Brown, Paul; Bransgore C Of E Primary School, Ringwood Rd, Bransgore, Christchurch BH23 8JH<br>Sancho, Michelle; West Berkshire Council, Council Offices, Market St, Newbury RG14 5LD<br>Creswell , Cathy ; University of Oxford,
**Primary Subject Heading**:	Mental health
Secondary Subject Heading:	Evidence based practice, Paediatrics, Qualitative research
Keywords:	Anxiety disorders < PSYCHIATRY, Protocols & guidelines < HEALTH SERVICES ADMINISTRATION & MANAGEMENT, MENTAL HEALTH, Child & adolescent psychiatry < PSYCHIATRY, QUALITATIVE RESEARCH

SCHOLARONE™
Manuscripts

I, the Submitting Author has the right to grant and does grant on behalf of all authors of the Work (as defined in the below author licence), an exclusive licence and/or a non-exclusive licence for contributions from authors who are: i) UK Crown employees; ii) where BMJ has agreed a CC-BY licence shall apply, and/or iii) in accordance with the terms applicable for US Federal Government officers or employees acting as part of their official duties; on a worldwide, perpetual, irrevocable, royalty-free basis to BMJ Publishing Group Ltd ("BMJ") its licensees and where the relevant Journal is co-owned by BMJ to the co-owners of the Journal, to publish the Work in this journal and any other BMJ products and to exploit all rights, as set out in our licence.

The Submitting Author accepts and understands that any supply made under these terms is made by BMJ to the Submitting Author unless you are acting as an employee on behalf of your employer or a postgraduate student of an affiliated institution which is paying any applicable article publishing charge ("APC") for Open Access articles. Where the Submitting Author wishes to make the Work available on an Open Access basis (and intends to pay the relevant APC), the terms of reuse of such Open Access shall be governed by a Creative Commons licence – details of these licences and which Creative Commons licence will apply to this Work are set out in our licence referred to above.

Other than as permitted in any relevant BMJ Author's Self Archiving Policies, I confirm this Work has not been accepted for publication elsewhere, is not being considered for publication elsewhere and does not duplicate material already published. I confirm all authors consent to publication of this Work and authorise the granting of this licence.

For peer review only

Protocol for the co-design and development of a primary school-based pathway for child anxiety screening and intervention delivery: A mixed-methods feasibility study

Victoria, Williamson.[a,b*], Michael Larkin.[c] , Tessa Reardon[a] , Samantha Pearcey[a], Claire Hill[d], Paul Stallard[e], Susan H Spence[f], Maria Breen[d], Ian Macdonald[g], ObiohaUkoumunne[n,], Tamsin Ford[h], Mara Violato[i], Falko Sniehotta[j], Jason Stainer[k], Alastair Gray[i], Paul Brown[l], Michelle Sancho[m], Cathy Creswell. [a]

[a] Department of Experimental Psychology, Anna Watts Building, University of Oxford, Oxford, OX2 6 GG

[b]Institute of Psychiatry, Psychology and Neuroscience, King's College London, London, SE5 9RJ

[c] Aston Neuroscience Institute, Department of Psychology, Aston University, Birmingham, B4 7ET

[d] School of Psychology & Clinical Language Sciences, University of Reading, Reading, RG6 6AL

[e] University of Bath, Claverton Down, Bath BA2 7AY

[f] Griffith University, 16 Russell Street South Bank, QLD 4101, AU

[g] Charlie Waller Memorial Trust, First Floor, Rear Office, 32 High Street, Thatcham RG19 3JD

[h] Department of Psychiatry, University of Cambridge, Cambridge, UK

[I] Nuffield Department of Population Health, University of Oxford, Richard Doll Building, Old Road Campus, Oxford OX3 7LF | UK

[j] Population Health Science Institute, Faculty of Medical Sciences, Newcastle University, Baddiley-Clark Building, Richardson Road, NE2 4AX

For peer review only - http://bmjopen.bmj.com/site/about/guidelines.xhtml

[k] Stanley Primary School, Strathmore Road, Teddington, Middlesex, TW11 8UH

[l] Bransgore C Of E Primary School, Ringwood Rd, Bransgore, Christchurch BH23 8JH

[m] West Berkshire Council, Council Offices, Market St, Newbury RG14 5LD

[n] NIHR ARC South West Peninsula, University of Exeter, Stocker Rd, Exeter EX4 4PY

*Correspondence: Dr Victoria Williamson, Department of Experimental Psychology, University of Oxford, Oxford, OX2 6 GG; Institute of Psychiatry, Psychology and Neuroscience, King's College London, London, SE5 9RJ. Victoria.williamson@kcl.ac.uk

Word Count: 3944

For peer review only

For peer review only - http://bmjopen.bmj.com/site/about/guidelines.xhtml

Abstract

Introduction: Anxiety difficulties are among the most common mental health problems in childhood. Despite this, few children access evidence-based interventions and school may be an ideal setting to improve children's access to treatment. This paper describes the design, methods, and expected data collection of the Identifying Child Anxiety Through Schools – Identification to Intervention (iCATS i2i) study which aims to develop acceptable school-based procedures to identify and support child anxiety difficulties.

Methods and analysis: iCATS i2i will use a mixed-methods approach to co-design and deliver a set of procedures – or 'pathway' – to improve access to evidence-based intervention for child anxiety difficulties through primary schools in England. The study will consist of four stages, initially involving in-depth interviews with parents, children, school staff, stakeholders (Stage 1) to inform the development of the pathway. The pathway will then be administered in two primary schools, including screening, feedback to parents, and the offer of treatment where indicated (Stage 2), with participating children, parents and school staff invited to provide feedback on their experience (Stages 3 & 4). Data will be analysed using Template Analysis.

Ethics and dissemination: The iCATS i2i study was approved by the University of Oxford's Research Ethics Committee (REF R64620/RE001). It is expected that this co-design study will lead on to a future feasibility study and, if indicated, a randomised controlled trial. The findings will be disseminated in several ways, including via lay summary report, publication in academic journals and presentation at conferences. By providing information on child, parent, school-staff and other stakeholder's experiences, we anticipate that the findings will inform the development of an acceptable evidence-based pathway for identification and intervention for children with

For peer review only - http://bmjopen.bmj.com/site/about/guidelines.xhtml

anxiety difficulties in primary schools and may also inform broader approaches to screening for and treating youth mental health problems outside of clinics.

Strengths and limitations of this study:

- Focus on child anxiety difficulties, one of the most common mental helath problems in childhood.
- By using a co-design approach which incorporates feedback from children, parents, school-staff and stakeholders, this study will lead to the development of acceptable procedures for screening and offering treatment for child anxiety difficulties in primary schools.
- The study is limited by the use of an 'opt-in' approach to consent which could introduce participation bias.
- The primary use of online platforms for consent, screening and delivery of the CBT intervention may exclude families who have limited access to technology or lack technical skills; although ways to facilitate the participation of those in these situations will be explored.

For peer review only

Introduction

Anxiety difficulties are among the most common mental health problems in childhood (6.5% prevalence; [1]) and approximately half of all anxiety difficulties emerge by the age of 11 years. Childhood anxiety difficulties are often chronic and pervasive, and have an adverse effect on social, education and familial functioning. Childhood anxiety difficulties often persist into adulthood when left untreated [2] and are associated with comorbid mental health difficulties, including major depression and substance abuse [3,4]. The societal cost of a child with an anxiety difficulty is estimated to be 21 times that of a non-anxious child [5]. As such, effective identification and treatment of anxiety difficulties in childhood is important.

Effective treatments for childhood anxiety exist. However, very few children are offered or are able to access them [6,7]. For example, previous research has shown that only 2% of pre-adolescent children who meet criteria for an anxiety disorder in England receive an evidence-based intervention [7]. Barriers to receiving evidence-based treatment can include problems with the identification of anxiety difficulties, concerns regarding stigma to the child or family, as well as a scarcity of trained mental health professionals and long waiting lists for specialist services [8,9]. Practically speaking, attending group or face-to-face programs can also bring logistical barriers for parents with young families including time demands, and difficulties with arranging transportation or child care [10–12].

The vast majority of children attend and spend much of their time at school, therefore schools are also an ideal setting to overcome many of these barriers [12,13]. However, there is not a clear set of procedures for identifying youth mental health difficulties and promoting access to evidence-based treatments in schools. Moreover, previous international studies have found mixed support for school based screening

For peer review only - http://bmjopen.bmj.com/site/about/guidelines.xhtml

and interventions for childhood anxiety, with some studies reporting reductions in child anxiety symptoms [12,14], while other studies have not [15]. Furthermore, some studies have reported low uptake to school-based interventions, for reasons including parents finding screening questionnaires too time consuming, parent concerns about stigma, as well as fears that their child may become more anxious from having had to discuss their worries [14]. This highlights the need for novel approaches to promote school based approaches to increase access to early intervention for childhood anxiety difficulties that are acceptable and well tolerated in order to to increase parent participation.

One approach often used in healthcare service design and development is 'co-design', where the knowledge and lived experiences of service users themselves are drawn upon to enhance the quality and experiences of care [16,17]. Co-design aims to develop an in-depth understanding of how stakeholders and service users perceive and experience the look, feel, procedures and structures of a service [18]. By engaging stakeholders and service users in co-designing a service, this is thought to lead to better quality of care and improved service performance by highlighting individual's subjective experiences at various points in the care pathway, which, in turn, may lead to improvements in health outcomes and more efficient allocation of limited healthcare resources [19]. Given the importance of early intervention, an acceptable school-based pathway that incorporates the identification of children with anxiety difficulties and promotes uptake of evidence-based intervention is urgently needed. The Identifying Child Anxiety Through Schools – Identification to Intervention (iCATS i2i) study will develop procedures to identify and support child anxiety difficulties through schools informed by a co-design approach.

For peer review only - http://bmjopen.bmj.com/site/about/guidelines.xhtml

This article describes the iCATS i2i co-design protocol. Data collection for this study will take place between December 2019 - December 2020. The co-designed procedures will be evaluated in a subsequent feasibility study and, if indicated, randomised control trial beginning in 2021.

Method

This protocol and associated procedures were approved by the Central University Research Ethics Committee at the University of Oxford (REF R64620/RE001).

Study design

We will apply a mixed-methods approach to co-design, produce and deliver a set of procedures – or 'pathway' - to improve access to evidence-based intervention for child anxiety difficulties through primary schools (i.e. ages 5-11) in England. Several of the key elements of the pathway were specified in advance of the co-design work based on the existing empirical literature and parent and school staff consultation. Specifically, it was pre-specified that children's anxiety difficulties would be screened using questionnaire measures, parents would receive feedback, and, where indicated, a brief, parent-led online intervention for child anxiety difficulties would be offered. The delivery of online treatment directly to parents was included as it has potential to overcome many of the barriers to care described above, such as logistical issues for parents and parental concerns about negative impacts on the child of participating in treatment [7,8].

In parallel to this work we are working on refining measures for screening for child anxiety problems (Reardon et al. under review), but in the interim we will screen using brief child, parent and teacher versions of the Spence Child Anxiety Scale (SCAS-8;[20]) together with four items that assess the extent of interference in everyday life

For peer review only - http://bmjopen.bmj.com/site/about/guidelines.xhtml

(e.g. "Do fears and worries stop you from doing things?") generated to assess the impact and chronicity of and perceived need for help for anxiety difficulties. The addition of interference items is known to improve the efficacy of similar self-report measures [21]. We will consider a child to have screened 'positive' for likely anxiety difficulties if they score above the cut off on the SCAS-8 on the basis of any reporter (score of 7.5 for parents, 6.5 for children, 4.5 for teachers) and/or indicate that anxiety interferes at least 1 'only a little' on any of the interferance items. This interference-based cut off score was based on feedback from the dedicated stakeholder group. The use of a screening questionnaire to determine which children may benefit from additional support with anxiety was a prespecified component of the study as this approach shows promise for increasing access to support (e.g. [22]).

The intervention to be offered is an online version of a brief therapist-guided parent-delivered cognitive behavioural therapy (CBT) approach for child anxiety difficulties (OSI; Online Support and Intervention for child anxiety). OSI was originally developed for use in National Health Service (NHS) clinics and was co-designed by NHS clinicians, parents and children who had received treatment for anxiety (Hill et al., in preparation). This intervention was selected as it is brief, effective [23] and more cost-effective than brief face-to-face psychological therapy [24] and can be delivered by non-expert practitioners (e.g. [25]). The approach of working directly with the parent, rather than the child, also addressed particular barriers to seeking and accessing help for anxiety highlighted by parents, including the preference to be supported to manage the difficulties as a family and for the child not to be singled out [7]. The online version of this intervention involves 7 online modules for parents, supported by a weekly 20-minute telephone call with a Children's Wellbeing Practitioner (CWP, NHS Band 5), with a follow-up telephone session 4-weeks later. Modules teach parents how to explore

For peer review only - http://bmjopen.bmj.com/site/about/guidelines.xhtml

their child's anxious thoughts, put them to the test through facing fears, and to problem solve challenges that arise. This is accompanied by a game app for the child to help motivate them to face their fears. The CWPs are postgraduate psychological therapists who have received specific (12 month) training in the delivery of brief psychological therapies for children and young people who have difficulties with anxiety, low mood, and behavioural disturbance. CWPs are based within settings where they can offer rapid access to psychological therapies, often including school based clinical services, and so are the ideal workforce to implement the approach being developed if indicated.

We will use a mixed-method co-design process to determine *how* the pre-specified parts of the pathway should be presented, who by, and to address any important considerations to optimise accessibility of and engagement with the pathway. The co-design process will consist of four stages (see Figure 1) involving initial interviews and focus groups with parents, children, school staff, stakeholders (Stage 1) to inform the development of a set of procedures that will be applied in two schools. These procedures will be delivered with participating children, parents and school staff (Stage 2) who will provide feedback on their experience (Stage 3 & 4), including cued-recall specifically on the experience of receiving feedback on whether their child experiences difficulties with anxiety. Feedback from those families who choose not to be involved in the study or dropped out will also be sought to ensure any barriers to engagement are captured (Stage 4).

Patient and public involvement (PPI)

Involvement from parents, school staff and wider stakeholders informed the development of this protocol, the pre-specified elements of the pathway and will contribute throughout the delivery of the co-design project. At the protocol development stage, consultation was carried out with parents, school staff/governors,

For peer review only - http://bmjopen.bmj.com/site/about/guidelines.xhtml

leading experts in universal screening and interventions in primary school settings, and representatives from key policy and practitioner organisations. Examples of decisions that were made on the basis of this consultation include specifically focussing recruitment on children in Year 4 (age 8-9 years) on the basis that this would be a manageable time for primary schools, would allow primary schools to see the benefit, and for children to benefit when managing subsequent key transitions (e.g. to secondary school).

Throughout the co-design process we will consult with stakeholders in the following ways: (i) two parents with relevant lived experience, two school leaders and one mental health lead for a charity are members of the study management group and will contribute to all decisions made at a strategic level; (ii) this dedicated stakeholder group will meet to review data and to make decisions to address how to solve problems and manage potentially conflicting points of view that have arisen through the co-design process; and (iii) a distinct, separate online PPI group will also be formed, made up primarily of parents. Members will be invited to join via the circulation of adverts about the online group (e.g. advert shared on social media, circulation of advert to parents from participating Stage 2 schools), with the purpose of the group being to access wider parental views about study procedures and on key issues that arise during the study.

Co-design Participants

Participants will include Y4 children (aged 8-9 years), parents of Y4 children, primary school staff and other stakeholders. Expected participant numbers for each group and at each stage in the co-design process are outlined in Table 1. These numbers are approximate and final numbers will be informed by reviewing the range of perspectives represented in the sample and the information provided by participants.

For peer review only - http://bmjopen.bmj.com/site/about/guidelines.xhtml

To recruit participants with a broad range of perspectives to Stage 1 we will circulate study invitations to parents of all Year 4 children in two primary schools in the local Oxfordshire area, as well as using online on social media, and mailing lists to recruit parents with particular experiences. For the subsequent stages we will first contact school leaders to invite their school to participate, and circulate study information to Y4 parents and children inviting them to participate.

All adult participants will be required to give written consent and children will be required to give written assent to participate in all stages of the project.

Inclusion criteria:

Children will be eligible to participate if they are in Y4 in a mainstream primary school in England, with parent/carer consent for their participation (Stages 1-4).

Parent/carers of children in Y4 in mainstream primary schools in England will be eligible to take part in Stages 1-4. However for Stage 1, we will also recruit parents through other routes in order to capture a range of experiences that might be particularly relevant to parents' engagement with and the accessibility of the pathway procedures, specifically parents who have a child with past/present mental health problem(s) or who is adopted/fostered, or where a parent has past/present mental health problem(s), or is in the military (due to their experience of frequent relocations, extended separation from parents, and parental physical or psychological injuries [26]).

School staff will be included if they are employed in a mainstream primary/junior school in England (e.g. class teacher, headteacher) (Stages 1-4).

The inclusion criteria for wider stakeholders is that they must be a member of an organisation that is responsible for policy or practice relating to mental health provision in primary schools in England (e.g. commissioning group, local authority,

For peer review only - http://bmjopen.bmj.com/site/about/guidelines.xhtml

mental health service provider, local policy maker organisation, or a governor in mainstream primary/junior schools) (Stage 1 and 4).

Table 1

Recruitment estimates for the co-design

Assessment	Planned N			
	Stakeholders	Teachers	Parents	Children
Stage 1. Initial focus groups	2	7	16	9
Stage 2. Administering procedures			144	144
Stage 3. Cued recall interviews		4	12	
Stage 4. Post-screening interviews/focus groups	12	12	12	12

Procedure

We will collect data at four stages to inform the development of the pathway (see Figure 1).

Stage 1. In this stage we will carry out in-depth one-to-one interviews and focus groups with stakeholders, school staff, children and parents. Focus groups and interviews will

For peer review only - http://bmjopen.bmj.com/site/about/guidelines.xhtml

draw on questioning techniques informed by the Critical Incident Approach [27] to explore participants' views about features of the pathway which might help or hinder a positive experience, or which might have been overlooked by the pathway planners altogether. Focus groups and interviews will include table-top activities where participants are shown visual representations of different aspects of the pathway and they will be asked to discuss and write on provided notecards which will be placed on the table about their thoughts, feelings and concerns, with questions including: "What would be the best way to do this?", "Who do you think would be best placed to do this?", "What might need to be done to help this part happening?","Where would be the best place for this to happen?","When is the best time to do this?", "Do you have any concerns about this part of the pathway?". Photographs will be taken of the visuals produced in the focus groups and interviews for analysis. Interviews and focus groups will be audio-recorded and transcribed verbatim. This data will be used to develop a detailed prototype set of procedures for screening, feedback and intervention delivery through schools to be tested and developed further. The dedicated stakeholder group will be consulted at key decision making points in the process to generate solutions to problems raised or inconsistent messages elicited from the interviews.

Stage 2. The detailed prototype set of procedures developed in Stage 1 will be administered in two primary schools, including screening, feedback to parents, and the offer of early intervention where indicated. We will encourage each school to nominate a member of staff to be the 'pathway lead' (e.g. a class teacher, the school's pastoral lead) who will be given training and psychoeducation by the research team about childhood anxiety difficulties and the proposed pathway procedures. The 'pathway lead' will coordinate recruitment efforts at their participating school, such as circulating study information sheets amongst Y4 parents. During Stage 2, we will quantitatively

For peer review only - http://bmjopen.bmj.com/site/about/guidelines.xhtml

examine pathway outcomes, including the proportion of parents in Y4 who agree to participate in the screening, the number of eligible parents who take up the intervention, the number of parents who withdraw, and symptom improvement rates. We will also conduct interviews with school staff, children and parents (including children who screened 'negative' and those who screened 'positive' for anxiety and their parents, and parents that did and did not take up the intervention) to examine their experiences of the pathway and potential barriers/facilitators to engagement.

Stage 3. On the basis of the dedicated stakeholder input at the protocol design stage, we anticipate that parents will be given written feedback on their child's screening outcomes by the school 'pathway lead', with the option of a face-to-face feedback appointment. The dedicated stakeholder group considered that feedback from the school 'pathway lead' would be preferred by families as families would likely have pre-existing relationships with the school and a member of school staff would therefore be well placed to introduce the CWP and the option to access the intervention. If this is supported by the outcomes of the earlier stages, the school staff member that is nominated to be the 'pathway lead' will receive training and guidance from the research team on delivering feedback to parents. To understand how this feedback is experienced, what works well and what parents (and the school staff 'pathway lead') find both helpful and challenging, participating parents and staff will be invited to take part in a cued-recall interview meeting to allow for the refinement of future feedback delivery and staff training. To this effect, the parent-staff feedback meetings will be video recorded by the research team. Recordings of the meeting will be watched back by parents with a member of the research team to facilitate discussions about how questionnaire scores were fed back and how the opportunity to take up the intervention was shared with parents by 'pathway lead' school staff member. The researcher will

For peer review only - http://bmjopen.bmj.com/site/about/guidelines.xhtml

invite the parent to stop the recording periodically to comment at points that are relevant to particular cues, for example, at points where the parent felt the information delivered was unhelpful, or where they felt listened to. The cued-recall interview will be audio-recorded and transcribed verbatim.

Stage 4. Following the administration of the pathway procedures, interviews and focus groups will also be carried out with Y4 children, their parents and school staff in Stage 4. We will carry out interviews with participating parents and children who completed the screening questionnaires and engage with the intervention modules, but also with any parents and children who withdraw and parents and children who choose not to enrol in the study. School staff (e.g., the 'pathway lead', Y4 class teachers) in participating schools will be interviewed about their experience of facilitating the pathway in Y4. Relevant stakeholders will also be interviewed about their views of the pathway and how well it will fit within school settings.

Data analysis

Focus groups and one-to-one interviews (Stages 1 and 4) will be analysed using two approaches: 'fast and direct' and 'in-depth and detailed'. The 'fast and direct' analysis will use the visual outputs from focus groups and interviews to collate themes and written summaries will provide readily understandable feedback about the pathway. Brief, complementary descriptions will be produced by following a simple protocol for verbalising 'multi- modal data' [28]The combination of thematised images and verbal summary will provide immediate, easily- understood feedback about the pathway.

The 'in-depth and detailed' analysis will involve Template Analysis [29] where an initial template is structured by categories drawn from relevant literature and further developed by preliminary coding of the data using a 'bottom up' approach. Once the template is developed, all transcripts will be analysed in a 'top down' manner

For peer review only - http://bmjopen.bmj.com/site/about/guidelines.xhtml

following the provisional structure of the template. This will provide nuanced feedback about the acceptability of the pathway to fine-tune the final iteration. This analysis will capture areas of disagreement that may be missed in the 'fast and direct' analysis. Cued-recall data (Stage 3) will also be analysed using Template Analysis [29]. Credibility will be checked via analytic triangulation using reflective discussions with co-analysts.

Ethics and dissemination

This research is being conducted in a community setting and ethical approval has been obtained from the University of Oxford's Research Ethics Committee. We will seek consent from parents, school staff and other stakeholders and assent from children. Research data will be kept secure and confidential. Audio/video-recordings will require explicit consent.

A key part of this project will involve developing acceptable procedures for feeding back outcomes from screening questionnaires which may bring potential to cause participant distress. Given that existing screening questionnaires have modest sensitivity and specificity, this includes explaining the possibility of an inaccurate result. We will pay particular attention to this throughout the co-design process to ensure we develop acceptable procedures and will seek out families who received 'false-positive' screening feedback to specifically explore their experience. Given the risk of 'false negatives', during this co-design phase the online intervention will be made available to all families who take part, along with information about additional resources, support and services.

Dissemination. This project aims to develop an effective pathway to identify child anxiety difficulties in mainstream schools and deliver a parent-led intervention through ongoing collaborative work with schools, parents, children and stakeholders, while fostering avenues for disseminating the results directly to the community.

For peer review only - http://bmjopen.bmj.com/site/about/guidelines.xhtml

Specifically, this co-design study will lead on to a future feasibility study and, if indicated, a randomised controlled trial. To disseminate the findings from this initial stage research, we will produce and disseminate a report that summarises outcomes in lay-language to participating schools, which will also be shared with participating families on request. The findings will be published in high-quality, open access journals that reach both academic, educational and clinical audiences. The research team will also present the findings at national and international clinical/educational conferences. We will also collaborate with our dedicated stakeholder group to further develop the dissemination plans to ensure maximum impact.

Discussion

There is currently no evidence-based pathway for identification and intervention for children with anxiety difficulties in primary schools. Despite the existence of cost-effective psychological treatments, very few children who could benefit are able to access them [7]. This project aims to generate knowledge using a co-design approach to inform the development of such a pathway that links screening with the direct provision of support for primary school aged children to ensure that it is acceptable and ultimately implementable within schools in England. By providing information on children, parents, school-staff and other stakeholder's experiences of this school-based pathway which includes screening for likely child anxiety difficulties, feedback on scores and the offer of an online intervention, we anticipate that the findings will also inform broader approaches to screening for and treating youth mental health problems outside of clinics.

This research has several methodological limitations that warrant consideration. First, because of the nature of child and parent involvement in this study, we will use an 'opt-in' approach to consent, where parents must consent to their and their child's

For peer review only - http://bmjopen.bmj.com/site/about/guidelines.xhtml

completion of the screening measures. This is likely to introduce bias in participation, and we risk failing to capture experiences of the pathway procedures for a sufficiently broad and diverse group of families where child anxiety difficulties (including for example, families who do not have concerns about child anxiety or where other barriers may exist, such as concerns about stigma). To address this, we will actively invite parents to Stage 1 and 4 interviews who both did and did not consent to screening as well as examine in interviews whether an 'opt-out' approach to screening would be acceptable in future iterations (e.g., screening measures are administered to the entire Y4 class unless parents opt-out their child from participating). A second potential limitation is that the study will primarily use online platforms for consent, and screening procedures, and to deliver the CBT intervention. This decision was informed by feedback from the dedicated stakeholder group who recommended that online participation was often considered more secure in terms of data protection and privacy. However, it introduces a risk of excluding families who have limited access to technology/Wi-Fi or lack technical skills or confidence. We will explore ways to enable participants in these situations to participate, and participant experiences of online access to the study will be examined.

With these potential limitations in mind, it is our intention that this study will collaboratively create a pathway to care for children who have problems with anxiety and their families, informed by children themselves, parents, school staff and other stakeholders, that will ultimately improve access to effective treatment and support.

Funding: This paper represents independent research funded by the National Institute for Health Research (NIHR; RP-PG-0218-20010) (CC). The views expressed are those of

For peer review only - http://bmjopen.bmj.com/site/about/guidelines.xhtml

the authors and not necessarily those of the NHS, the NIHR or the Department of Health and Social Care.

Competing interests: Authors have no conflicts of interest to declare.

Contributorship statement: All authors (Victoria, Williamson.[a,b*], Michael Larkin.[c] , Tessa Reardon[a] , Samantha Pearcey[a], Claire Hill[d], Paul Stallard[e], Sue Spence[f], Maria Breen[d], Ian Mcdonald[g], ObiohaUkoumunne[n,], Tamsin Ford[h], Mara Violato[i], Falko Sniehotta[j], Jason Stainer[k], Alastair Gray[i], Paul Brown[l], Michelle Sancho[m], Cathy Creswell. [a])

contributed towards the design of the study protocol, contributed towards the writing of the manuscript and approved the final version of the protocol. Authors (Victoria, Williamson.[a,b*], Michael Larkin.[c] , Tessa Reardon[a] , Samantha Pearcey[a], Sue Spence[f], Ian Mcdonald[g], Tamsin Ford[h], Jason Stainer[k], Paul Brown[l], Cathy Creswell. [a]) contributed towards data collection and analysis.

For peer review only

References

1 Polanczyk G V., Salum GA, Sugaya LS, *et al.* Annual research review: A meta-analysis of the worldwide prevalence of mental disorders in children and adolescents. *J Child Psychol Psychiatry Allied Discip* 2015;**56**:345–65. doi:10.1111/jcpp.12381

2 Hofstra MB, Van Der Ende J, Verhulst FC. Continuity and change of psychopathology from childhood into adulthood: A 14-year follow-up study. *J Am Acad Child Adolesc Psychiatry* 2000;**39**:850–8. doi:10.1097/00004583-200007000-00013

3 Adams GC, Balbuena L, Meng XF, *et al.* When social anxiety and depression go together: A population study of comorbidity and associated consequences. *J Affect Disord* 2016;**206**:48–54. doi:10.1016/j.jad.2016.07.031

4 Brook JS, Zhang C, Rubenstone E, *et al.* Comorbid trajectories of substance use as predictors of Antisocial Personality Disorder, Major Depressive Episode, and Generalized Anxiety Disorder. *Addict Behav* 2016;**62**:114–21. doi:10.1016/j.addbeh.2016.06.003

5 Bodden DHM, Dirksen CD, Bögels SM. Societal burden of clinically anxious youth referred for treatment: A cost-of-illness study. *J Abnorm Child Psychol* 2008;**36**:487–97. doi:10.1007/s10802-007-9194-4

6 Essau CA, Muris P, Ederer EM. Reliability and validity of the Spence Children's Anxiety Scale and the Screen for Child Anxiety Related Emotional Disorders in German children. *J Behav Ther Exp Psychiatry* 2002;**33**:1–18. doi:10.1016/S0005-7916(02)00005-8

7 Reardon T, Harvey K, Creswell C. Seeking and accessing professional support for child anxiety in a community sample. *Eur Child Adolesc Psychiatry* 2020;**29**:649–

For peer review only - http://bmjopen.bmj.com/site/about/guidelines.xhtml

64. doi:10.1007/s00787-019-01388-4

8 Mojtabai R, Olfson M, Sampson NA, *et al.* Barriers to mental health treatment: results from the National Comorbidity Survey Replication. *Psychol Med* 2011;**41**. doi:10.1017/S0033291710002291

9 Schnyder N, Sawyer MG, Lawrence D, *et al.* Barriers to mental health care for Australian children and adolescents in 1998 and 2013–2014. *Aust N Z J Psychiatry* 2020;**54**. doi:10.1177/0004867420919158

10 Axford N, Lehtonen M, Kaoukji D, *et al.* Engaging parents in parenting programs: Lessons from research and practice. *Child Youth Serv Rev* 2012;**34**:2061–71. doi:10.1016/j.childyouth.2012.06.011

11 Morgan AJ, Rapee RM, Salim A, *et al.* Internet-Delivered Parenting Program for Prevention and Early Intervention of Anxiety Problems in Young Children: Randomized Controlled Trial. *J Am Acad Child Adolesc Psychiatry* 2017;**56**:417-425.e1. doi:10.1016/j.jaac.2017.02.010

12 Mifsud C, Rapee RM. Early intervention for childhood anxiety in a school setting: Outcomes for an economically disadvantaged population. *J Am Acad Child Adolesc Psychiatry* 2005;**44**:996–1004. doi:10.1097/01.chi.0000173294.13441.87

13 Neil AL, Christensen H. Efficacy and effectiveness of school-based prevention and early intervention programs for anxiety. Clin. Psychol. Rev. 2009;**29**:208–15. doi:10.1016/j.cpr.2009.01.002

14 McLoone JK, Rapee RM. Comparison of an Anxiety Management Program for Children Implemented at Home and School: Lessons Learned. *School Ment Health* 2012;**4**:231–42. doi:10.1007/s12310-012-9088-7

15 Miller LD, Laye-Gindhu A, Liu Y, *et al.* Evaluation of a preventive intervention for child anxiety in two randomized attention-control school trials. *Behav Res Ther*

For peer review only

2011;**49**:315–23. doi:10.1016/j.brat.2011.02.006

16 Dimopoulos-Bick, Dawda P, Maher L, *et al.* Experience-Based Co-Design: Tackling common challenges | The Journal of Health Design. *J Heal Des* 2019;**3**:86–93.https://www.journalofhealthdesign.com/JHD/article/view/46 (accessed 15 Sep 2020).

17 Bevan Jones R, Stallard P, Agha SS, *et al.* Practitioner review: Co-design of digital mental health technologies with children and young people. J. Child Psychol. Psychiatry Allied Discip. 2020;**61**:928–40. doi:10.1111/jcpp.13258

18 Palmer VJ, Chondros P, Piper D, *et al.* The CORE study protocol: A stepped wedge cluster randomised controlled trial to test a co-design technique to optimise psychosocial recovery outcomes for people affected by mental illness in the community mental health setting. *BMJ Open* 2015;**5**:e006688. doi:10.1136/bmjopen-2014-006688

19 Boyd H, McKernon S, Mullin B, *et al.* Improving healthcare through the use of co-design. *N Z Med J* 2012;**125**.https://www.nzma.org.nz/journal-articles/improving-healthcare-through-the-use-of-co-design (accessed 15 Sep 2020).

20 Reardon T, Spence SH, Hesse J, *et al.* Identifying children with anxiety disorders using brief versions of the Spence Children's Anxiety Scale for children, parents, and teachers. *Psychol Assess* 2018;**30**:1342–55. doi:10.1037/pas0000570

21 Evans R, Thirlwall K, Cooper P, *et al.* Using symptom and interference questionnaires to identify recovery among children with anxiety disorders. *Psychol Assess* 2017;**29**:835–43. doi:10.1037/pas0000375

22 D'Souza CM, Forman SF, Austin SB. Follow-up evaluation of a high school eating disorders screening program: Knowledge, awareness and self-referral. *J Adolesc*

For peer review only

Heal 2005;**36**:208–13. doi:10.1016/j.jadohealth.2004.01.014

23 Thirlwall K, Cooper PJ, Karalus J, *et al.* Treatment of child anxiety disorders via guided parent-delivered cognitive-behavioural therapy: Randomised controlled trial. *Br J Psychiatry* 2013;**203**:436–44. doi:10.1192/bjp.bp.113.126698

24 Creswell C, Violato M, Fairbanks H, *et al.* Clinical outcomes and cost-effectiveness of brief guided parent-delivered cognitive behavioural therapy and solution-focused brief therapy for treatment of childhood anxiety disorders: a randomised controlled trial. *The Lancet Psychiatry* 2017;**4**:529–39. doi:10.1016/S2215-0366(17)30149-9

25 Thirlwall K, Cooper P, Creswell C. Guided parent-delivered cognitive behavioral therapy for childhood anxiety: Predictors of treatment response. *J Anxiety Disord* 2017;**45**:43–8. doi:10.1016/j.janxdis.2016.11.003

26 Williamson V, Stevelink SAM, Da Silva E, *et al.* A systematic review of wellbeing in children: a comparison of military and civilian families. *Child Adolesc Psychiatry Ment Health* 2018;**12**:46. doi:10.1186/s13034-018-0252-1

27 Butterfield LD, Borgen WA, Amundson NE, *et al.* Fifty years of the critical incident technique: 1954-2004 and beyond. *Qual Res* 2005;**5**:475–97. doi:10.1177/1468794105056924

28 Boden Z, Eatough V. Understanding More Fully: A Multimodal Hermeneutic-Phenomenological Approach. *Qual Res Psychol* 2014;**11**:160–77. doi:10.1080/14780887.2013.853854

29 Brooks J, McCluskey S, Turley E, *et al.* The Utility of Template Analysis in Qualitative Psychology Research. *Qual Res Psychol* 2015;**12**:202–22. doi:10.1080/14780887.2014.955224

For peer review only

For peer review only

For peer review only - http://bmjopen.bmj.com/site/about/guidelines.xhtml

Figure 1. Overview of the co-design process for developing the iCATS i2i protocol

Note. Y4 = year four.

For peer review only

For peer review only - http://bmjopen.bmj.com/site/about/guidelines.xhtml

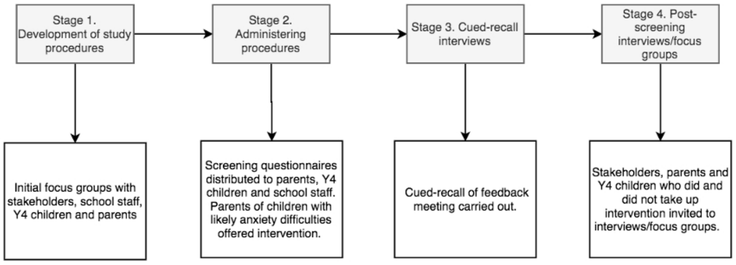

Figure 1. Overview of the co-design process for developing the iCATS i2i protocol

Note. Y4 = year four.

Figure 1
